# Fungal cytochrome P450 monooxygenases of *Fusarium oxysporum* for the synthesis of ω-hydroxy fatty acids in engineered *Saccharomyces cerevisiae*

**DOI:** 10.1186/s12934-015-0228-2

**Published:** 2015-04-02

**Authors:** Pradeepraj Durairaj, Sailesh Malla, Saravanan Prabhu Nadarajan, Pyung-Gang Lee, Eunok Jung, Hyun Ho Park, Byung-Gee Kim, Hyungdon Yun

**Affiliations:** School of Biotechnology, Yeungnam University, Gyeongsan, South Korea; School of Chemical and Biological Engineering, Seoul National University, Seoul, South Korea; Department of Bioscience and Biotechnology, Konkuk University, Seoul, South Korea; Current position: Novo Nordisk Foundation Center for Biosustainability, Technical University of Denmark, Copenhagen, Denmark

**Keywords:** Cytochrome P450, Cytochrome P450 reductase, Omega fatty acid hydroxylase, cDNA gene cloning, Heterologous expression, Saccharomyces cerevisiae

## Abstract

**Background:**

Omega hydroxy fatty acids (ω-OHFAs) are multifunctional compounds that act as the basis for the production of various industrial products with broad commercial and pharmaceutical implications. However, the terminal oxygenation of saturated or unsaturated fatty acids for the synthesis of ω-OHFAs is intricate to accomplish through chemocatalysis, due to the selectivity and controlled reactivity in C-H oxygenation reactions. Cytochrome P450, the ubiquitous enzyme is capable of catalyzing the selective terminal omega hydroxylation naturally in biological kingdom.

**Results:**

To gain a deep insight on the biochemical role of fungal P450s towards the production of omega hydroxy fatty acids, two cytochrome P450 monooxygenases from *Fusarium oxysporum* (*FoCYP*), *FoCYP539A7* and *FoCYP655C2;* were identified, cloned, and heterologously expressed in *Saccharomyces cerevisiae*. For the efficient production of ω-OHFAs, the *S. cerevisiae* was engineered to disrupt the acyl-CoA oxidase enzyme and the β-oxidation pathway inactivated (ΔPox1) *S. cerevisiae* mutant was generated. To elucidate the significance of the interaction of redox mechanism, *FoCYP*s were reconstituted with the heterologous and homologous reductase systems - *S. cerevisiae* CPR (*ScCPR*) and *F. oxysporum* CPR (*FoCPR*). To further improve the yield, the effect of pH was analyzed and the homologous *FoCYP-FoCPR* system efficiently hydroxylated caprylic acid, capric acid and lauric acid into their respective ω-hydroxy fatty acids with 56%, 79% and 67% conversion. Furthermore, based on computational simulations, we identified the key residues (Asn106 of *FoCYP539A7* and Arg235 of *FoCYP655C2*) responsible for the recognition of fatty acids and demonstrated the structural insights of the active site of *FoCYP*s.

**Conclusion:**

Fungal CYP monooxygenases, *FoCYP539A7* and *FoCYP655C2* with its homologous redox partner, *FoCPR* constitutes a promising catalyst due to its high regio- and stereo-selectivity in the hydroxylation of fatty acids and in the substantial production of industrially valuable ω-hydroxy fatty acids.

**Electronic supplementary material:**

The online version of this article (doi:10.1186/s12934-015-0228-2) contains supplementary material, which is available to authorized users.

## Background

Fatty acids (FA) are simple and indispensable molecules of all biological systems usually derived from triglycerides or phospholipids and exist as carboxylic acids with long unbranched saturated / unsaturated aliphatic chain molecules. The FAs are modified to generate hydroxy-, epoxy-, amino-, nitro-, and halogen- derivatives, which are building blocks for various complex molecules [1]. The hydroxylation of hydrocarbon occurring closer to the carboxyl group results in α- or β-hydroxylation, and in the terminal ending give rise to ω-hydroxylation. Terminally oxidized omega hydroxy fatty acids (ω-OHFAs) are multifunctional compounds employed in the production of various industrial products with broad commercial and pharmaceutical implications including adhesives, lubricants, cosmetic intermediates and potential anticancer agents [2,3]. ω-OHFAs derived from the medium or long chain fatty acids serve as building blocks for the synthesis of poly (ω-hydroxy fatty acids) and polymers like bioplastics with high water resistance, durability and chemical versatility [4,5], which demands the substantial increase in the production of various fatty acid derivatives [1,6]. ω-OHFAs are chemically procured by the cross-metathesis of unsaturated fatty acid esters, preceded by the hydroformylation and hydrogenation of the carbonyl group [7,8]. However, the terminal oxygenation of saturated or unsaturated fatty acids for the synthesis of ω-OHFAs is intricate to accomplish through chemo catalysis, due to the selectivity and controlled reactivity in C-H oxygenation reactions [4]. Besides, the chemical synthesis of ω-OHFAs is expensive due to the formation of various byproducts that demand substantial purification strategies and affect the sustainability as it relies on severe reaction conditions and high energy demanding processes [9].

In biological systems, selective omega hydroxylation occurs naturally in mammals, plants and in certain yeast and bacteria, mostly catalyzed by the cytochrome P450 (CYP) monooxygenases [10]. Cytochrome P450, the ubiquitous enzyme forms a vast divergent family of heme-thiolate proteins and performs a broad range of versatile enzymatic activities. The class II P450 enzymes along with their heme donor, cytochrome P450 reductase (CPR) execute hydroxylation of various endogenous and exogenous compounds and are involved in xenobiotic detoxification and degradation as well. Microbial cytochrome P450s are of great potential interest as they act as biocatalysts and are key elements not only for microbial natural product formation but also in bioremediation. In addition, they also play a major role as drug and agrochemical targets [11]. Cytochrome P450 enzymes are capable of catalyzing intricate reactions like regio- and stereo- selective oxidation of unactivated hydrocarbon C–H bonds to the corresponding hydroxy (C–OH) products [12]. These P450 enzymes are also accountable for the initial and rate limitings step of n-alkane and fatty acid hydroxylation [13]. Currently, the biosynthetic ω-OHFAs are produced by the members of microbial CYPs like CYP52 (P450Alk) and CYP153 through the selective terminal oxygenation of fatty acids. Multiple CYP52 genes have been identified in the yeast *Candida* species and they encode isozymes with different or overlapping substrate specificities [13]. Nevertheless immense progress has been accomplished, low space-time yields and biocatalyst recycling affects the industrialization of these processes, which ultimately paves the way for new biotechnological production strategies.

Although various omega hydroxylase P450 monooxygenases have been identified, there are no standard reports for omega hydroxylation in the filamentous fungal kingdom. Fungal genome sequencing projects have revealed the existence of more than 6000 fungal genes coding for putative P450s which are yet to be explored for the discovery of novel catalytic enzymes [13,14]. These fungal CYP enzymes indulge in the biosynthesis of a vast array of secondary metabolites of biomedical, agricultural, and industrial significance [15]. With the goal of developing an alternative fungal based process to produce beneficial ω-OHFAs, we investigated the novel CYPs from *Fusarium oxysporum* f.sp *lycopersici (Fol),* which is a well characterized; genome sequenced phyto-pathogenic fungi. In recent years, *Fol* also emerged as a mammalian pathogen by affecting immuno-compromised humans and mammals, and thus evolved as a dual plant-mammal infection system [16]. Among the genome sequenced *Fusarium* strains, *F. oxysporum* has the largest genome size (60 MB) comprising the greater number of protein-encoding genes (17,735) as compared to its most closely related species, *Fusarium graminearum* (13,332) and *Fusarium verticillioides* (14,179) [16]. Besides, *F. oxysporum* encompasses the unique bifunctional cytochrome P450s, CYP55A1 (P450nor) and CYP505A1 (P450foxy) [17,18]. Both P450nor and P450foxy are self-sufficient P450s; P450nor is very essential for fungal denitrification and P450foxy accounts for the ω-1 to ω-3 hydroxylation of fatty acids. *F. oxysporum* thus stands unique and signifies the molecular evolutionary path of cytochrome P450 by possessing eukaryotic CYPs with functional properties similar to those of prokaryotes.

**Figure 1:**
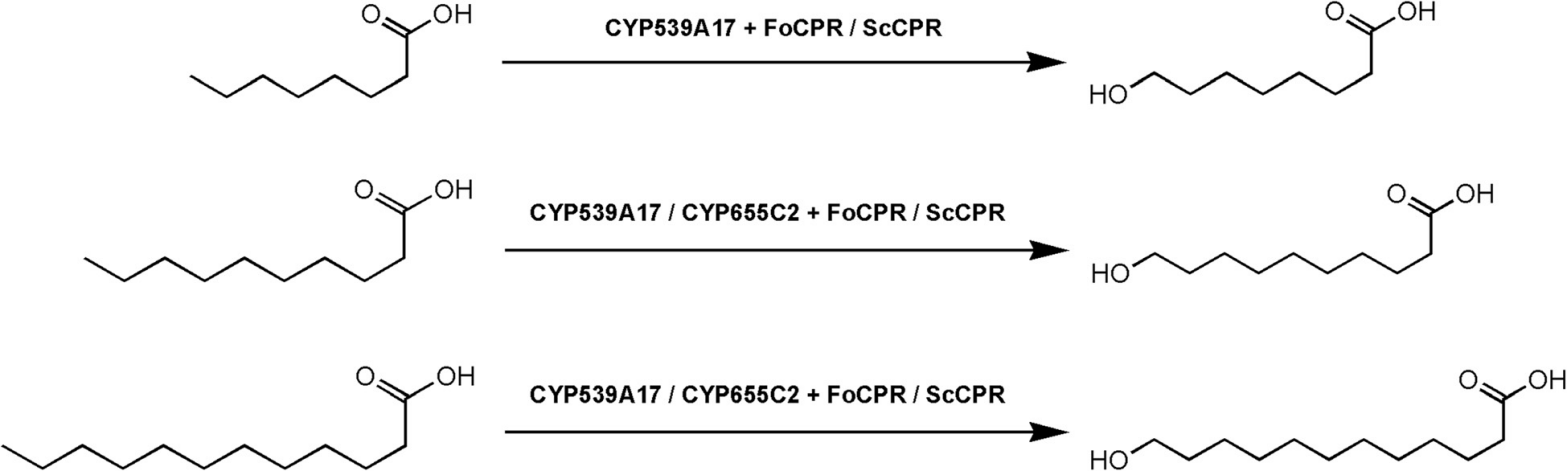
**Reaction scheme of omega hydroxylation of fatty acids by** ***Fusarium oxysporum*** **cytochrome P450 monooxygenases (** ***FoCYP*** **) with the heterologous (** ***ScCPR*** **) and homologous (** ***FoCPR*** **) reductases.** *FoCYP539A7* can hydroxylate caprylic acid (C8), capric acid (C10) and lauric acid (C12) into their respective ω-hydroxy fatty acids, whereas *FoCYP655C2* can hydroxylate only capric acid and lauric acid.

To gain a deep insight into the biochemical role of fungal P450s in the production of omega hydroxy fatty acids, we selected two cytochrome P450 monooxygenases from *F. oxysporum* (*FoCYP*), *FoCYP539A7* and *FoCYP655C2,* and heterologously expressed them in *Saccharomyces cerevisiae*. For the efficient production of ω-OHFAs, the *S. cerevisiae* was engineered to disrupt the acyl-CoA oxidase enzyme and the β-oxidation pathway inactivated (ΔPox1) *S. cerevisiae* mutant was generated. The *FoCYP*s were reconstituted with the heterologous and homologous reductases -*S. cerevisiae* CPR (*ScCPR*), *Candida albicans* CPR (*CaCPR*) and *F. oxysporum* CPR (*FoCPR*) to elucidate the significance of the redox mechanism. Comparative analysis of the differential redox partners with the *FoCYP*s revealed the enhanced production and broader substrate specificity of *FoCYP539A7* with *FoCPR*. Withal, molecular modeling studies were performed to demonstrate the structural insights of the active site of *FoCYP*s. To the best of our knowledge, this is the first report demonstrating the comparative analysis of heterologous and homologous reductases with the fungal omega hydroxylase cytochrome P450 monooxygenases in the synthesis of ω-OHFAs (Figure 1).

## Results and discussion

### Gene selection and sequence analysis of *FoCYP539A7* and *FoCYP655C2*

*Fusarium oxysporum* stands distinct and intrigued the noteworthy attraction for functional characterization by not only encompassing the bifunctional CYPs, P450nor and P450foxy, but also due to the inclusion of larger pool of other cytochrome P450 genes. The *insilico* analysis of *Fusarium oxysporum* f.sp *lycopersici* genome based on the Fungal Cytochrome P450 Database [19] revealed the presence of 169 putative cytochrome P450s suggesting that *Fol* has unique metabolic processes that are predominantly involved in both primary and secondary metabolism. To identify the ω-fatty acid hydroxylase monooxygenases among the 169 putative CYPs of *F. oxysporum* (*FoCYP*), phylogenetic analysis was carried out with the reported ω-selective or ω-specific fatty acid hydroxylases (CYP52) of *Candida* species [12]. The phylogenetic tree generated by the neighbor-joining method showed the presence of 6 putative *FoCYP*s within the same gene cluster of reported CYP52 family, signifying the likelihood of sharing the conserved P450 motifs such as distal helices and substrate recognition sites towards ω-FA hydroxylation (Additional file 1: Figure S1). We aimed to functionally characterize all 6 putative *FoCYP*s, but only the FOXG_00101, FOXG_14594 and FOXG_03506 gene candidates were amplified from the cDNA generated from the RNA cocktail mixture obtained from different day cultures of *F. oxysporum*. The FOXG_14589, FOXG_10811 and FOXG_03951 candidates were not amplified in both enriched (PDA) and minimal (nitrogen limited) medium even after repeated attempts, probably due to the lack of mRNA expression. Genomic sequence analysis revealed that FOXG_03506 gene candidate is not a full length P450; hence the FOXG_00101 and FOXG_14594 were subjected to functional characterization. According to Nelson’s classification system, although the P450s act on the fatty acid substrates, they are classified into different CYP families based on their amino acid identity [20]. Dr.Nelson’s Cytochrome P450 Database [20] has classified and designated the FOXG_00101 and FOXG_14594 candidates into the P450 superfamily as CYP539A7 and CYP655C2, respectively; and hence they are represented as *FoCYP539A7* and *FoCYP655C2* in this manuscript. Multiple sequence alignment analysis of *FoCYP539A7* and *FoCYP655C2* with the CYP52 candidates revealed the sequence similarities and showed the typical heme binding domain FNAGPRICIG and FGGGPRRCPA; respectively, in the C terminal region (Additional file 1: Figure S2). The sequence identity of *FoCYP539A7* was found to be 42% towards CYP52A9 [21], CYP52A13 [22], CYP52A17 and CYP52A21 [23], 41% towards CYP52A3 [21] and CYP52A4 [21], and 40% towards CYP52A5 [21]. Correspondingly, the sequence identity of *FoCYP655C2* was found to be 32% towards CYP52A9 and CYP52A21, 31% towards CYP52A13, CYP52A17 and CYP52A3 and 30% towards CYP52A4 and CYP52A5. The homologous nature of the *FoCYP539A7* and *FoCYP655C2* with the CYP52 family suggests the likeliness of structural and enzymatic functions towards ω-FA hydroxylation.

### Heterologous expression and functional characterization of *FoCYP*s in *S. cerevisiae*

**Figure 2:**
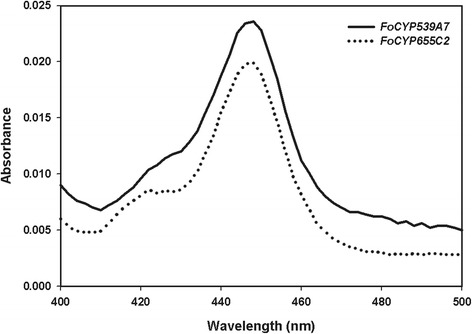
**CO Binding analysis of microsomes of** ***FoCYP539A7*** **and** ***FoCYP655C2*** **expressed in** ***S. cerevisiae.*** The solid line represents *FoCYP539A7* and the dotted line represents *FoCYP655C2.* Yeast expression was carried out in *S. cerevisiae* cells using 4% galactose, 2 mM 5-ALA at 30°C.

For the heterologous expression of eukaryotic CYPs and for the extensive enzyme production and synthesis of value added chemicals, yeast system is the preferred host because of the presence of an endoplasmic reticulum membrane environment and the combination of higher eukaryotic protein machinery [24-28]. Hence, we aimed to heterologously express full-length *FoCYP539A7* and *FoCYP655C2 genes* encoding 533 and 512 amino acid residues directly in the yeast *S. cerevisiae* BY4742 cells. The amplified *FoCYP* genes were cloned into pESC-URA vectors and designated as pU-FoCYP539A7 and pU-FoCYP655C2 in this manuscript (Additional file 1: Figure 1A). The pU-FoCYP539A7 and pU-FoCYP655C2 vector constructs were transformed into the *S. cerevisiae* cells individually and to elucidate its heterologous expression, microsomes were isolated and CO difference spectral analysis was carried out. The reduced CO-difference spectral analysis carried out with the yeast microsomes of *FoCYP539A7* and *FoCYP655C2* resulted in an absorption maximum at 448 nm confirming the active P450 nature (Figure 2). Based on the CO-difference spectra, the concentration of the isolated microsomes of *FoCYP539A7* and *FoCYP655C2* were estimated to be 0.189 nmol/mL and 0.176 nmol/mL respectively, and the active P450 obtained from a 500 mL yeast culture were 0.378 nmol and 0.352 nmol respectively. CO-binding analysis performed with the microsomes of the *S. cerevisiae* cells harboring only the pESC-URA plasmid without *FoCYP* did not show any peak around 450 nm, which confirmed the successful expression of active *FoCYP539A7* and *FoCYP655C2,* and also demonstrated the lack of interference of intrinsic yeast CYPs due to their low levels of expression.

The sole functional activity of CYPs depends mainly on their accessory protein partner CPR for the electron transfer from NADPH to the heme group of CYPs. The NADPH reductase from yeast is a highly efficient and prominent redox donor for transferring electrons to various heterologous CYPs. To compare the interference of CPR over the catalytic efficiency of *FoCYP*s, the well reported yeast NADPH reductases from *S. cerevisiae* (*ScCPR*) [29] and *C. albicans* (*CaCPR*) [30] were employed. The *ScCPR* and *CaCPR* reductase genes encoding 691 and 680 amino acid residues amplified from the respective genomic DNA were cloned into the pESC-LEU vector and designated as pL-ScCPR and pL-CaCPR, respectively (Additional file 1: Figure 1B). The CPR vector constructs was transformed and reconstituted individually into the yeast *S. cerevisiae* cells harboring pU-FoCYP539A7 and pU-FoCYP655C2 for co-expression and functional analysis. The yeast reconstituted system harboring pU-FoCYP539A7 and pL-ScCPR/pL-CaCPR gene constructs were termed CYP539A7-ScCPR and CYP539A7-CaCPR respectively in this manuscript. Similarly, the yeast reconstituted systems harboring the pU-FoCYP655C2 and pL-ScCPR/pL-CaCPR gene constructs were termed CYP655C2-ScCPR and CYP655C2-CaCPR respectively. Initially, the substrate specificity and functional catalytic efficiency of *FoCYP539A7* and *FoCYP655C2* reconstituted systems were analyzed both in an *in vitro* system and in a resting cell system with the medium and long chain fatty acids: lauric acid (C12), myristic acid (C14) and palmitic acid (C16) using 100 μM substrate concentration. Microsomes were isolated from all the reconstituted systems of *S. cerevisiae* cells and the *in vitro* reactions were performed with the standard assay mixture. Upon incubation, the products were extracted and derivatized with BSTFA for gas chromatographic analysis. However, we were not able to observe any quantifiable data in GC analysis, probably due to the instability of the microsomal proteins and the low expression levels of the fungal cytochrome P450 systems. Subsequently, the resting cell reaction was carried out with galactose induced reconstituted systems (as mentioned above) of *S. cerevisiae* cells (~400 mg/mL) in both Tris-HCl and potassium phosphate buffer (pH 7.0) with 2% dextrose or galactose. Nevertheless, GC analysis of the trimethylsilylated reaction samples did not show any significant substrate consumption or product formation in any of the reconstituted systems. This could be possibly due to the fact that the P450 being an unstable enzyme, it might have degraded during the enzyme reaction or perhaps the NADPH required for the monooxygenase reaction was not sufficient enough to produce any catalytic conversion.

To overcome this, the growing whole cell (biotransformation) system was employed, since the growing cells permit less stable enzymes like cytochrome P450 to be expressed sustainably [28]. Biotransformation was carried out with the *S. cerevisiae* cells harboring CYP539A7-ScCPR, CYP539A7-CaCPR, CYP655C2-ScCPR and CYP655C2-CaCPRsystems, which were induced with 4% galactose with 2 mM 5-ALA. C12, C14 and C16 fatty acids were added to the growing cells in 500 μM substrate concentrations and the pH of the culture was continually maintained at pH 7.0 throughout the reaction. In the biotransformation carried out with long chain fatty acids (LCFA) such as myristic acid and palmitic acid, GC analysis of the trimethylsilylated reaction samples did not show any substrate consumption or product formation in any of the reconstituted systems. Interestingly, the biotransformation reaction samples of lauric acid in the CYP539A7-ScCPR and CYP655C2-ScCPR reconstituted systems showed significant substrate consumption, suggesting the possible involvement of *FoCYP*s with medium chain fatty acids (MCFA). However, no substrate consumption was observed in the case of CYP539A7-CaCPR and CYP655C2-CaCPR reconstituted systems probably due to the lack of compatibility of *CaCPR* with the *FoCYPs.* Correspondingly, no significant changes were obtained in the biotransformation carried out with the *S. cerevisiae* cells harboring only pU-FoCYP539A7 and pU-FoCYP655C2 constructs (control), signifying the lack of interference of intrinsic endogenous reductase with the fungal *FoCYP*s. Thus, the substrate consumption obtained in the CYP539A7-ScCPR and CYP655C2-ScCPR could be expounded as the result of catalytic reaction of *FoCYP*s with the *ScCPR*. To verify the stability of ω-OHFAs in *S. cerevisiae* BY4742 cells, ω-hydroxy lauric acid was fed to yeast systems harboring only pU-FoCYP539A7 and pU-FoCYP655C2 constructs (control) and cultured. The GC analysis of the 48 hr culture samples did not show any product peak elucidating that ω-OHFAs might have degraded naturally by the yeast.

### Construction of ΔPox1 mutant *S. cerevisiae* and synthesis of ω-OHFAs

**Figure 3:**
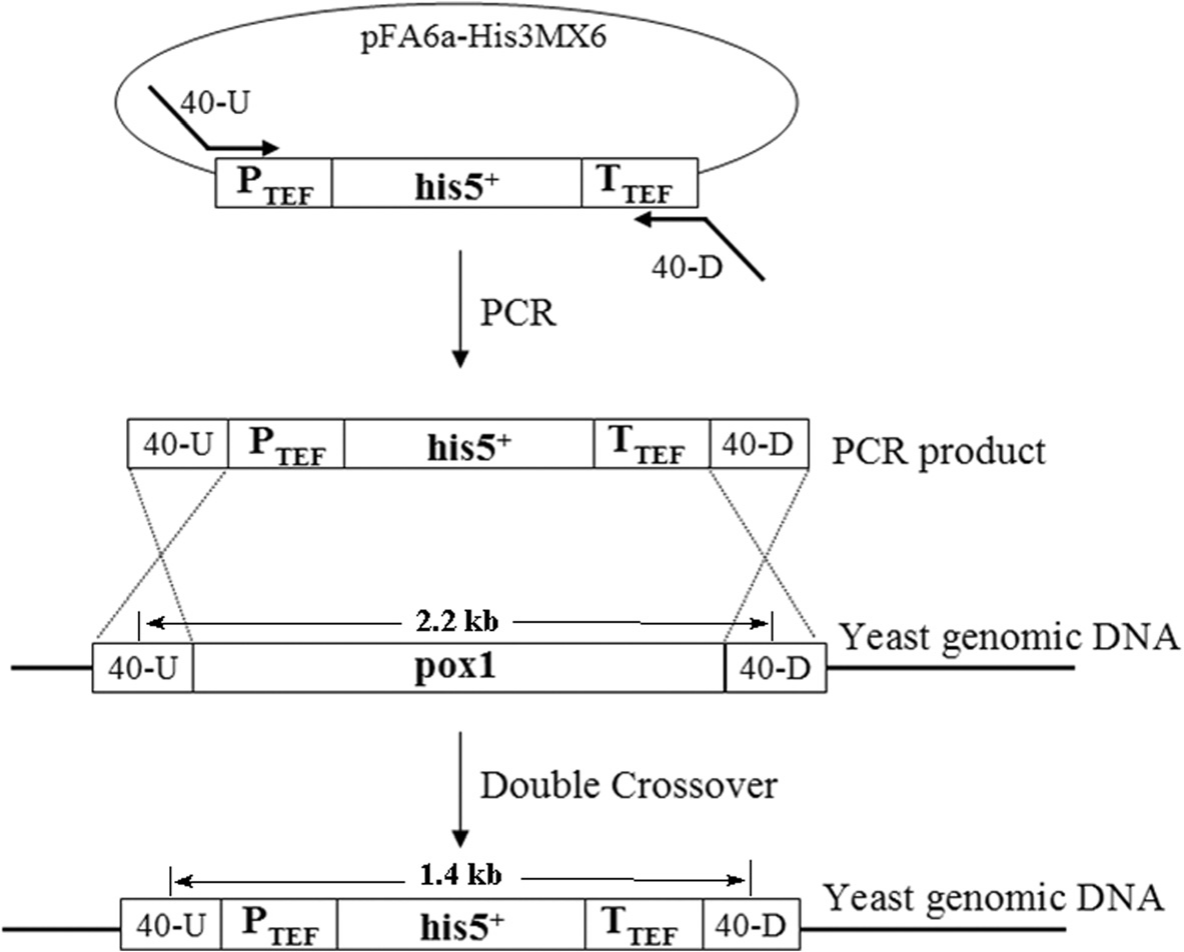
**Schematic representation of the strategy used to disrupt** ***pox1*** **gene of** ***S. cerevisiae*** **INV** ***S*** **c1 by PCR-mediated short-region homologous recombination.** The HisMX cassette was used to replace the *pox1*gene. The double alleles of *pox1* are replaced by the HisMX auxotrophic marker through homologous recombination

It is indispensable to consider the fact that in yeast systems the exogenously supplied fatty acids could be degraded in two different oxidation pathways: ω-oxidation in endoplasmic reticulum and β-oxidation in peroxisomes [26,31] (Additional file 1: Figure S4). The major constraint in yeast cell factory is that ω-oxidation is an alternative pathway to the β-oxidation, which becomes prominent when the latter is defective [2,32]. In the biotransformation carried out with the CYP539A7-ScCPR and CYP655C2-ScCPR systems, the ω-hydroxylated lauric acid could have degraded by the β-oxidation pathway of yeast cells, resulting in no product peak in the GC analysis. This provoked us to inactivate the β-oxidation pathway in the *S. cerevisiae* cells for better substrate availability to the heterologously expressed P450 enzymes and for the stability of hydroxylated fatty acids. The β-oxidation process is primarily comprised of four enzymes: acyl-CoA oxidase, enol-CoA hydratase, 3-hydroxy acyl-CoA dehydrogenase and 3-oxoacyl-CoA thiolase. The first and rate-limiting enzyme in this pathway is acyl-CoA oxidase, which is encoded by a single copy gene *pox1* in *S. cerevisiae* (Additional file 1: Figure S4). Sequential gene disruption of the acyl-CoA oxidase enzymes results in the functional blockage of the β-oxidation pathway thereby preventing the yeasts from utilizing fatty acids as a carbon source for cell growth. Inactivation of β-oxidation pathway thus becomes an attractive strategy in the metabolic engineering of yeast for the efficient production of ω-OHFAs from renewable sources [33]. Using PCR-mediated gene disruption technique, we deleted the chromosomal *pox1* from *S. cerevisiae* INV*Sc*1 for the most efficient blockage of the β-oxidation pathway and the *pox1* disrupted mutant was named *S. cerevisiae* ΔPox1 (Figure 3). Upon PCR amplification, only 1.4 kb sized gene band was obtained from the mutant strains, which confirmed the deletion of chromosomal *pox1* gene (Additional file 1: Figure S5). Notwithstanding, the development or engineering of the expression host is a prerequisite for the significant improvement in the production yields of ω-OHFA.

**Figure 4:**
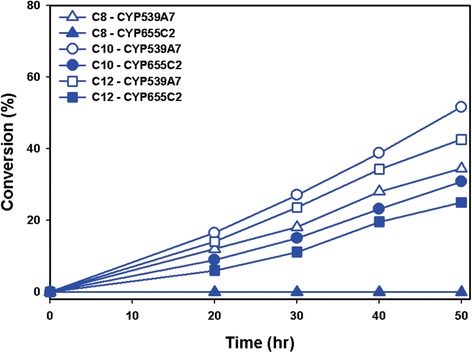
**Reaction profiles of hydroxylation of fatty acids by** ***FoCYP539A7*** **and** ***FoCYP655C2*** **with the heterologous (** ***ScCPR*** **) reductase.** ΔPox1 mutant *S. cerevisiae* cells harboring the CYP39A7-ScCPR and CYP655C2-ScCPR reconstituted systems were induced with 4% galactose, 2 mM 5-ALA and 500 μM of substrates: caprylic acid (C8), capric acid (C10) and lauric acid (C12) were added and cultured at pH 7.0. Samples collected at 10 hr intervals were extracted, trimethylsilyl derivatized and analyzed by GC.

**Figure 5:**
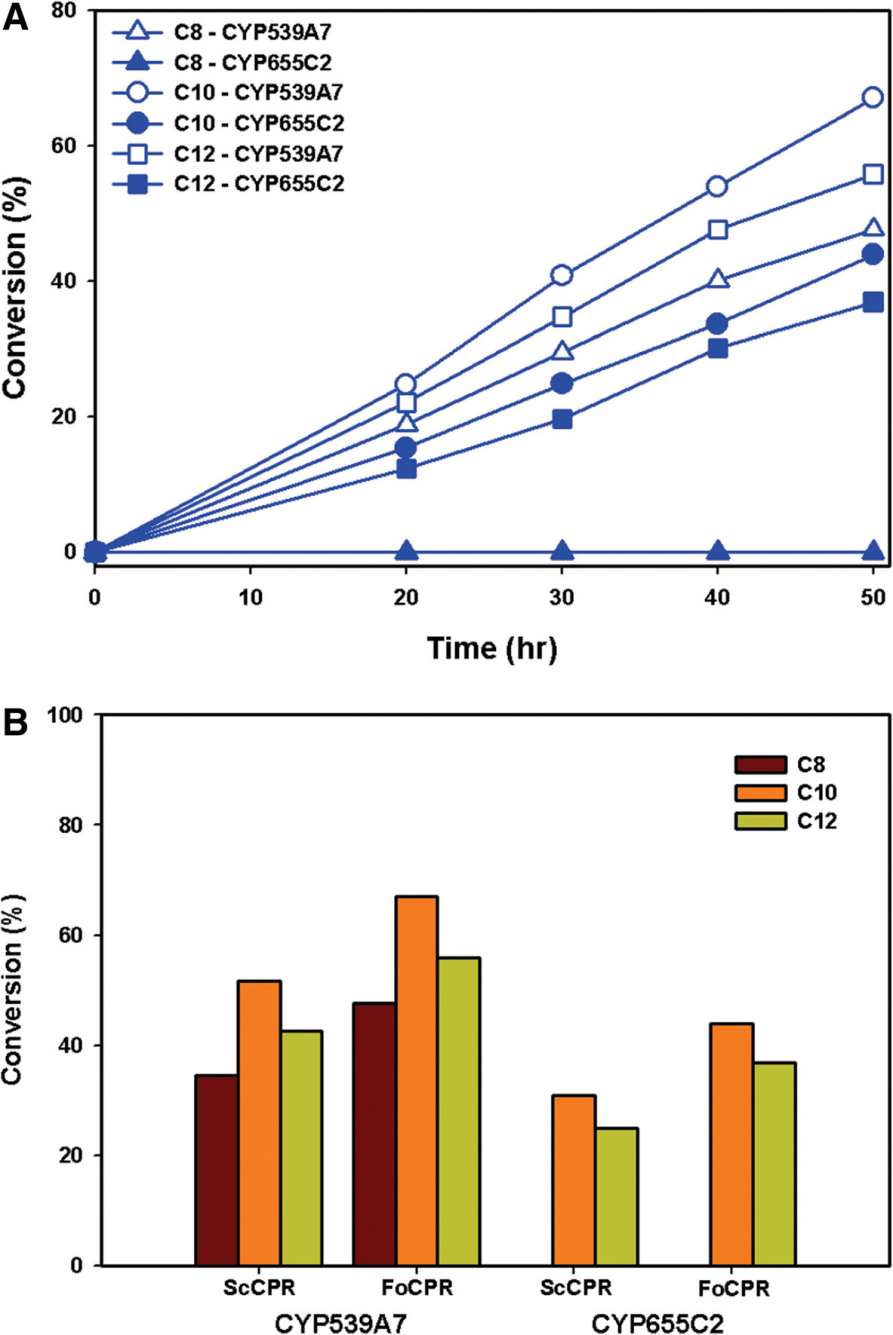
**5 Significance of homologous FoCYP-FoCPR reconstituted system in the hydroxylation of fatty acids. (A)** Reaction profile of hydroxylation of fatty acids by FoCYP539A7 and FoCYP655C2 with the homologous (FoCPR) reductase. **(B)** Comparative analysis on the catalytic conversion of fatty acids by FoCYP539A7 and FoCYP655C2 with the heterologous (ScCPR) and homologous (FoCPR) reductases. Data were plotted from the 50 hr biotransformation reaction samples. ΔPox1 mutant S. cerevisiae cells harboring the CYP539A7-FoCPR, CYP655C2-FoCPR, CYP39A7-ScCPR and CYP655C2-ScCPR reconstituted systems were induced with 4% galactose, 2 mM 5-ALA and 500 μM of substrates: caprylic acid (C8), capric acid (C10) and lauric acid (C12) were added and cultured at pH 7.0. Samples collected at 10 hr intervals were extracted, trimethylsilyl derivatized and analyzed by GC.

The pU-FoCYP539A7 and pU-FoCYP655C2 vector constructs were retransformed and reconstituted individually into the ΔPox1 mutant *S. cerevisiae* cells along with the pL-ScCPR for co-expression and functional analysis. GC analysis of the trimethylsilylated biotransformation samples of CYP539A7-ScCPR and CYP655C2-ScCPR reconstituted systems showed hydroxylation of lauric acid into ω-hydroxy lauric acid with 42.6% and 24.9% conversion (Figure 4). The significant hydroxylation of lauric acid by the *FoCYP539A7* and *FoCYP655C2* enzymes stimulated us to examine the other MCFAs including caproic acid (C6), caprylic acid (C8) and capric acid (C10). Interestingly, *FoCYP539A7* was active to both caprylic acid and capric acid*,* whereas *FoCYP655C2* showed activity only towards capric acid. CYP539A7-ScCPR reconstituted system hydroxylated capric acid into ω-hydroxy capric acid showing better conversion than lauric acid with 51.7% conversion (Figure 4) and hydroxylated caprylic acid into ω-hydroxy caprylic acid with 34.5% conversion (Figure 4). The CYP655C2-ScCPR reconstituted system showed only the hydroxylation of capric acid with 30.8% conversion (Figure 4). The eukaryotic fungal CYPs, *FoCYP539A7* and *FoCYP655C2* enzymes thus demonstrated their selective reactivity towards medium chain fatty acid hydroxylation (Figure 5B and Additional file 1: Table S1). The *S. cerevisiae* ΔPox1 mutant harboring *FoCYP* reconstituted systems significantly prevented the oxidation of ω-OHFAs to acetyl CoA due to the inactivation of the β-oxidation pathway.

### Significance of homologous *FoCYP-FoCPR* reconstituted system

In addition to the abundance of CYP, the sole monooxygenase reaction also relies on the abundance and electron transfer compatibility of its redox partner, CPR [34,35]. Hence, to maximize the redox coupling efficiency of P450 enzymes, co-expression with an appropriate functional CPR is crucial to achieve optimal CYP activity. For the efficient functional characterization of eukaryotic P450 genes, the homologous CYP-CPR system promotes enhanced monooxygenase activity due to their high electron transfer compatibility and coupling efficiency [34-36]. The reductase gene of *F. oxysporum* (*FoCPR*) and its paralogues were selected from the *Fusarium* comparative database [16] and examined in our study. In addition to the larger number of P450 genes, filamentous fungi like *F. oxysporum* encompass multiple CPR paralogues including FOXG_08274, FOXG_03206, FOXG_07461 and FOXG_04834 [37]. Sequence analysis of *F. oxysporum* CPR paralogues revealed that FOXG_08274 shared a high sequence identity with the reported CPR family compared to others. We intended to employ FOXG_08274 and FOXG_07461 CPR paralogues for the functional comparative analysis, but the mRNA pertaining to FOXG_07461 was not expressed in both enriched (PDA) and minimal (nitrogen limited) medium. However, multiple sequence alignment analysis of FOXG_08247 showed the FMN-, FAD-, and NADPH- binding domains to be well conserved and homologous with the reported CPR family. Hence, the full-length *FoCPR* (FOXG_08247) gene encoding 692 amino acid residues amplified from the *Fol* cDNA was cloned into the pESC-LEU vector and designated as pL-FoCPR (Additional file 1: Figure 3B). We attempted to construct a yeast reconstituted system of *FoCYP539A7* and *FoCYP655C2* with its homologous CPR to compare and scrutinize its functional activity and hence the newly generated reconstituted systems were termed CYP539A7-FoCPR and CYP655C2-FoCPR respectively. Gas chromatographic analysis of the biotransformation samples of CYP539A7-FoCPR system showed significant increase in the hydroxylation of caprylic acid, capric acid and lauric acid with 47.6%, 67.05% and 55.8% conversion, respectively (Figure 5A and B). Similarly, the CYP655C2-FoCPR system showed increased conversion of capric acid and lauric acid with 43.9% and 36.9% respectively (Figure 5A and B). The homologous *FoCYP-FoCPR* reconstituted system showed substantial improvement in the catalytic efficiency of both *FoCYP539A7* and *FoCYP655C2* enzymes (Figure 5B and Additional file 1: Table S1).

The differences in the bioconversion of fatty acid substrates between the heterologous and homologous reconstituted systems could possibly be due to the natural compatibility of *FoCYP*s towards the redox partner or due to the differences in the expression levels of P450 and CPRs [38]. Hence, parameters including the expression levels of both *FoCYP539A7* and *FoCYP655C2,* and the redox donors *ScCPR* and *FoCPR* in all the reconstituted systems were analyzed. Microsomes were isolated from the *S. cerevisiae* cells harboring CYP539A7-ScCPR, CYP539A7-FoCPR, CYP655C2-ScCPR and CYP655C2-FoCPR, and the total microsomal protein concentrations were calculated by bradford assay. Based on CO-binding analysis, the concentration of P450 in the CYP539A7-ScCPR and CYP539A7-FoCPR reconstituted systems were 0.115 nmol/mL and 0.137 nmol/mL respectively (Additional file 1: Figure S6A), while the CYP655C2-ScCPR and CYP655C2-FoCPR reconstituted systems had P450 concentrations of 0.081 nmol/mL and 0.112 nmol/mL respectively (Additional file 1: Figure S6B). Due to the possibility of loss of some fraction of P450 during the isolation procedure, the amount of P450 in the isolated microsomes was normalized based on the total microsomal protein concentration. The specific amounts of P450 in the microsomes containing CYP539A7-ScCPR, CYP539A7-FoCPR, CYP655C2-ScCPR and CYP655C2-FoCPR reconstituted systems were estimated to be 1.8, 1.85, 1.4 and 1.6 μmol of P450/mg of total protein respectively, demonstrating that the expression level of P450s in all the reconstituted systems was similar. Further, to compare the expression level of CPRs, we carried out the MTT reduction assay, where MTT (tetrazolium salt) was used as a substrate to measure the reduction activity of all co-expressed CPRs [39,40]. Equal amounts of total microsomal protein (10 μg/mL) of each reconstituted system were treated with MTT and the color change was observed following the addition of NADPH (Additional file 1: Figure S6C). Microsomes containing only *FoCYP539A7* and *FoCYP655C2* did not show any color change due to their inability to reduce MTT in the absence of CPR. The reduction of MTT into blue formazon was measured at 610 nm and an extinction coefficient of 11.3 mM^−1^ cm^−1^ was used to calculate the number of moles of MTT reduced. The rate of reduction of MTT by microsomes containing CYP539A7-ScCPR, CYP539A7-FoCPR, CYP655C2-ScCPR and CYP655C2-FoCPR were 10.01 μM/min, 10.2 μM/min, 9.9 μM/min and 9.5 μM/min respectively (Additional file 1: Figure S6D). The MTT reduction rate demonstrates that the expression levels of heterologous and homologous reductases in all the reconstituted systems were in the same range. Despite the fact that the residue sites pertaining to the substrate specificity reside in the active site of the P450, interaction of the CPR also plays a role in the outcome of CYP reactions [41]. Therefore, it can be derived that the variation in the catalytic efficiency of *FoCYP539A7 and FoCYP655C2* between the heterologous and homologous reconstituted systems is due to the interaction of CYP-CPR coupling efficiency and the electron transfer compatibility. The source of the reductase thus played a crucial role in the efficiency of the coupled reaction mediated by cytochrome P450 in terms of ω-OHFAs production. Hence, the functional activity of *FoCYP*s is highly influenced and administered by its homologous redox partner, *FoCPR*.

### Influence of pH on bioconversion

**Figure 6:**
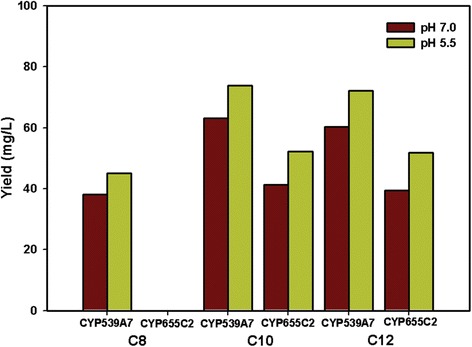
**Final yield (mg/L) of ω-hydroxy fatty acids by** ***FoCYP539A7*** **and** ***FoCYP655C2*** **with the homologous reductase (** ***FoCPR*** **) in the biotransformation carried out at pH 5.5 and pH 7.0.** Data were plotted from the 50 hr biotransformation reaction samples. ΔPox1 mutant *S. cerevisiae* cells harboring the CYP539A7-FoCPR and CYP655C2-FoCPR reconstituted systems were induced with 4% galactose, 2 mM 5-ALA and 500 μM of substrates: caprylic acid (C8), capric acid (C10) and lauric acid (C12) were added and cultured at pH 5.5 and pH 7.0. Samples collected at 10 hr intervals were extracted, trimethylsilyl derivatized and analyzed by GC.

To determine the influence and effect of pH on the bioconversion process, the pH of the growing whole cell reactions was continually adjusted to 5.5, the optimal pH for *S. cerevisiae* cell growth. It is noteworthy that the quantitative analysis of the biotransformation reaction carried out in pH5.5 showed a significant increase in the rate of product formation. The homologous CYP539A7-FoCPR and CYP655C2-FoCPR reconstituted systems showed increased hydroxylation of capric acid with 78.5% and 55.5% conversion, lauric acid with 66.7% and 51.5% conversion and caprylic acid with 56.1% conversion (Figure 6 and Additional file 1: S7A). Similarly the heterologous CYP539A7-ScCPR and CYP655C2-ScCPR reconstituted systems also showed increased hydroxylation of capric acid with 61.4% and 40.9% conversion, lauric acid with 55.4% and 38.4% conversion and caprylic acid with 45.3% conversion (Additional file 1: Figure S7B and S8). The pH 5.5, being an optimal condition for *S. cerevisiae* cell growth, could possibly indulge in the enhanced production of heterologously expressed P450 enzymes thereby favoring better product formation (Additional file 1: Table S1). Besides to verify the influence of pH, ω-hydroxy fatty acids were fed to the ΔPox1 mutant *S. cerevisiae* cells harboring only *FoCYP* without CPR (control) in both pH 5.5 and pH 7.0 culture conditions and the 24 hr samples were extracted and analyzed by GC. Interestingly, the pH 5.5 culture sample retained about 81.6% ω-OHFA, whereas pH 7.0 culture samples retained only 72.3%, probably due to the degradation or consumption of ω-OHFAs. The enhanced stability of ω-OHFAs in pH 5.5 could be presumed as the fact behind the increased production of ω-OHFAs by both *FoCYP539A7* and *FoCYP655C2* enzymes irrespective of the reductase systems (Figure 6, S8 and Additional file 1: Table S1). The order of conversion efficiency of fatty acids into their respective omega hydroxy fatty acids by *FoCYP539A7* is C10 > C12 > C8 and *FoCYP655C2* is C10 > C12. Overall, the CYP539A7-FoCPR reconstituted system showed better ω-OHFAs production compared to other reconstituted systems, signifying that *FoCYP539A7* with *FoCPR* is the better candidate in terms of substrate specificity and product formation (Figure 6 and Additional file 1: Table S1).

In addition, the trimethylsilylated metabolites were analyzed by GC-MS to qualitatively analyze the hydroxylated product. In the biotransformation with caprylic acid as a substrate, the hydroxylated TMS derivatized product displayed a mass spectrum with prominent ions at m/z 306, 290 (M-15, loss of CH_3_^**˙**^), 274 (M-31, loss of –CH_4_ and –CH_3_^**˙**^), 199 (M-105, loss of TMSOH-CH_3_^**˙**^), 147 [Me_2_Si = O^+^SiMe_3_], 145 [HO^+^ = C(-CH = CH_2_)-OSiMe_3_], 132 [CH_2_ = C(-OH)-OSiMe_3_], 129 [CH_2_ = CH-C(=O)-O^+^ = SiMe_2_] and 117 [CH_2_ = C(-OH)-O^+^ = SiMe_2_] and was identified as 8-hydroxyoctanoic acid (Additional file 1: Figure S9A and S10A). With capric acid as a substrate, the hydroxylated TMS derivatized product showed a mass spectrum with prominent ions at m/z 333, 318 (M-15, loss of CH_3_^**˙**^), 302 (M-31, loss of –CH_4_ and –CH_3_^**˙**^), 228 (M-105, loss of TMSOH-CH_3_^**˙**^), 217 [CH_2_ = CH-C(=O^+^SiMe_3_)-OSiMe_3_], 204 [CH_2_^**˙**^-C^+^(-OSiMe_3_)-OSiMe_3_], 147 [Me_2_Si = O^+^SiMe_3_], 145 [HO^+^ = C(-CH = CH_2_)-OSiMe_3_], 132 [CH_2_ = C(-OH)-OSiMe_3_], 129 [CH_2_ = CH-C(=O)-O^+^ = SiMe_2_] and 117 [CH_2_ = C(-OH)-O^+^ = SiMe_2_] and was identified as 10-hydroxydecanoic acid (Additional file 1: Figure S9B and S10B). When lauric acid was used as a substrate, the hydroxylated TMS derivatized product showed a mass spectrum with prominent ions at m/z 361, 346 (M-15, loss of CH_3_^**˙**^), 330 (M-31, loss of –CH_4_ and –CH_3_^**˙**^), 256 (M-105, loss of TMSOH-CH_3_^**˙**^), 217 [CH_2_ = CH-C(=O^+^SiMe_3_)-OSiMe_3_], 204 [CH_2_^**˙**^-C^+^(-OSiMe_3_)-OSiMe_3_], 147 [Me_2_Si = O^+^SiMe_3_], 145 [HO^+^ = C(-CH = CH_2_)-OSiMe_3_], 132 [CH_2_ = C(-OH)-OSiMe_3_], 129 [CH_2_ = CH-C(=O)-O^+^ = SiMe_2_] and 117 [CH_2_ = C(-OH)-O^+^ = SiMe_2_] and was identified as 12-hydroxydodecanoic acid (Additional file 1: Figure S9C and S10C). The ions at m/z 204 and 217 are formed via a trimethylsilyl transfer between the ether and the ester group. The MS patterns of the reaction metabolites were found to be identical to the respective standard compounds. Thus, both *FoCYP539A7* and *FoCYP655C2* reconstituted systems hydroxylated fatty acids at their ω-positions and produced ω-OHFAs demonstrating them to be omega hydroxylase monooxygenases (Figure 1).

### Molecular modeling studies

**Figure 7:**
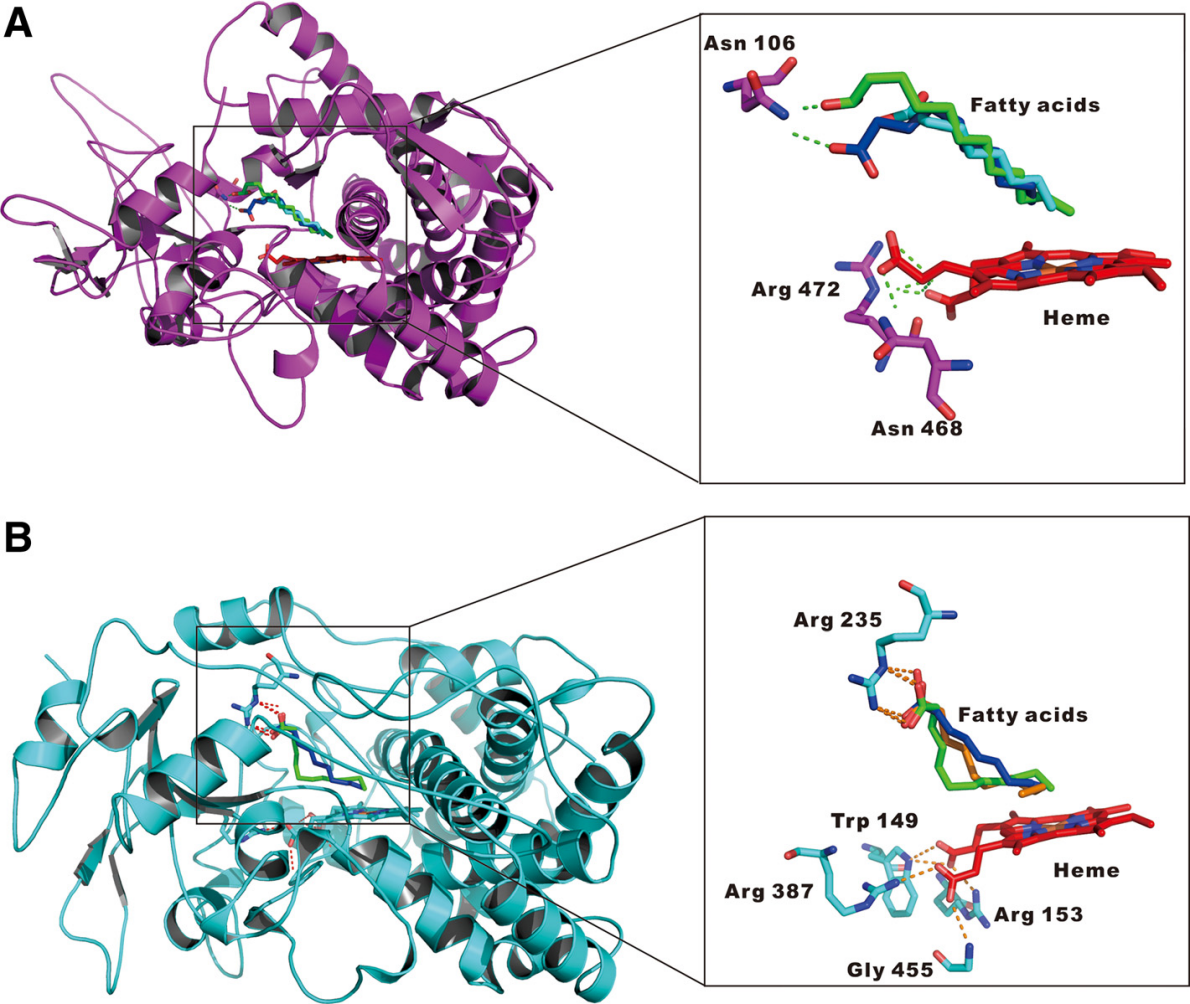
**Superimposition of docked complexes of fatty acids in the active site of** ***FoCYP*** **s. (A)** Superimposition of docked complexes of caprylic acid (cyan stick), capric acid (blue stick), and lauric acid (green stick) in the active site of *FoCYP539A7* (pink ribbons). Fatty acids show hydrogen bond interaction with Asn106 of *FoCYP539A7* and the ω carbon faces towards the ferric atom of heme. **(B)** Superimposition of docked complexes of capric acid (blue stick), lauric acid (green stick) and myristic acid (orange sticks) in the active site of *FoCYP655C2* (cyan ribbons). Fatty acids show hydrogen bond interaction with Arg235 of *FoCYP539A7* and the ω carbon faces towards the ferric atom of heme except myristic acid. The oxygen and nitrogen are represented in red and blue, and heme is represented as red sticks.

Although a large number of cytochrome P450s have been reported, the 3D structure, active site information and interaction of most of the cytochrome P450s with substrates remain unclear [42,43]. In this study, we predicted the model structures of *FoCYP539A7* and *FoCYP655C2* and their interactions with fatty acid substrates were analyzed to get the structural insight of CYP reactivity. It is reported that CYP undergoes conformational changes in the active site after substrate binding [44-46]. So, here we modeled the 3D structure of *FoCYP*s based on the heme domain using the best templates obtained through homology search against Protein databank. The *FoCYP539A7* model structure was constructed along with the heme structure using the template of *Homo sapiens* CYP co-crystallized with cholesterol-3-sulfate (PDB id - 2Q9F) [44] that shares 29% sequence identity (Additional file 1: Figure S11A&B and S12). Similarly, the *FoCYP655C2* was also constructed with heme using the template of *Homo sapiens* (PDB id - 1TQN) [46] that shares 27% sequence identity (Additional file 1: Figure S13A&B and S14). Initially, flexible docking was carried out with its best substrate capric acid (C10) to determine the key residues responsible for the hydrogen bond interaction of our modeled *FoCYP*s. From the docking study, it is clear that the Asn106 of *FoCYP539A7* is the key interacting amino acid to form hydrogen bond interaction with the carboxylic acid atom of capric acid (Figure 7A). This interaction helps the precise orientation of capric acid in the active sites of *FoCYP539A7* and favors the omega carbon atom to face towards the ferric atom of heme, thereby favoring omega hydroxylation. Similarly*,* Arg235 plays the key role in *FoCYP655C2* to form hydrogen bond interaction with the carboxylic acid moiety of capric acid (Figure 7B). Based on the screening, the active site pocket of both *FoCYP539A7* and *FoCYP655C2* residing near 5Å of docked capric acid was comprised with hydrophobic amino acids [Additional file 1: Table S2]. Further, docking of other fatty acid substrates such as C6, C8, C12 and C14 were carried out and the docked complexes favoring the similar hydrogen bond interaction as that of capric acid were exported and analyzed. In *FoCYP539A7,* the docked complexes of caprylic acid (C8), capric acid (C10) and lauric acid (C12) shared the same kind of interaction and orientation (Figure 7A) and the gold scores were 31.190, 31.5764 and 32.54, respectively. Unlike *FoCYP539A7,* only capric acid and lauric acid shared the same kind of orientation with *FoCYP655C2* (Figure 7B), and the gold scores were 48.3749 and 46.0965, respectively. Due to their shorter chain lengths, C6 and C8 fatty acids lack the normal hydrophobic interaction with the active site residues. In contrast, the C14 fatty acid failed to show the same kind of interaction and had a different orientation due to the presence of steric hindrance between the longer chain and the heme (Figure 7B). The docking results of *FoCYP539A7* and *FoCYP655C2* were well correlated with our experimental results in terms of substrate specificity and bioconversion. Based on this study, we can employ further site directed or specific mutagenesis in the active site residues of *FoCYP*s to extend the broad range of substrates and to increase the catalytic conversion of fatty acids.

## Conclusion

The first omega fatty acid hydroxylase CYP monooxygenases from *F. oxysporum* was successfully identified, cloned, heterologously expressed in the β-oxidation pathway inactivated (ΔPox1) *S. cerevisiae* mutant. Herein, we report the comparative study on the significance of heterologous and homologous CPRs in terms of functional catalytic activity of *FoCYP*s*.* The homologous CYP539A7-FoCPR and CYP655C2-FoCPR reconstituted systems produced 73.8 mg/L and 52.2 mg/L of 10-hydroxydecanoic acid, 72.2 mg/L and 51.9 mg/L of 12-hydroxydodecanoic acid, and 45.1 mg/L of 8-hydroxyoctanoic acid. Correspondingly, the heterologous CYP539A7-ScCPR and CYP655C2-ScCPR reconstituted systems produced 57.8 mg/L and 38.5 mg/L of 10-hydroxydecanoic acid, 56.8 mg/L and 36.0 mg/L of 12-hydroxydodecanoic acid, and 36.2 mg/L of 8-hydroxyoctanoic acid. *FoCYP539A7* and *FoCYP655C2* with their homologous redox partner, *FoCPR* constitutes a promising catalyst due to its high regio- and stereo-selectivity in the substantial production of industrially valuable ω-hydroxy fatty acids. In addition, we demonstrated the structural insights of active site of *FoCYP*s and the key residues (Asn106 of *FoCYP539A7* and Arg235 of *FoCYP655C2*) responsible for the recognition of fatty acids based on the computational simulations. Comprehensive studies are under progress to increase the substrate specificity and production of ω-OHFAs, and to elucidate the homologous redox coupling mechanism in the *FoCYP* mediated reactions. Subsequently, the results obtained in this study will pave the way for further biotechnological prospects to explore and exploit the novel catalytic properties of other *FoCYP*s.

## Methods

### Chemicals

All commercial chemicals including fatty acids and ω-hydroxy fatty acids (C6-16), 5-aminolevulinic acid (5-ALA), amino acids were purchased from Sigma (St. Louis, MO) or Aldrich Chemical Co. (Milwaukee, WI). N,O-Bis(trimethylsilyl)-trifluoroacetamide (BSTFA) was obtained from Fluka (Buchs, Switzerland). Ethyl acetate and dimethyl sulfoxide (DMSO) were purchased from Junsei (Japan) and Duksan (Ansan, Korea) respectively. Potato dextrose (PD) media, yeast peptone dextrose (YPD) media, yeast nitrogen base w/o amino acids and luria bertani (LB) media were purchased from BD Difco (Franklin Lakes, NJ). All chemicals used were of analytical grade.

### Microorganism and culture conditions

The fungal strain *Fusarium oxysporum* f. sp. *Lycopersici* strain 4287 was obtained from the Fungal Genetic Stock Centre (USA). The fungus was cultured on potato dextrose agar (PDA) for 4-5 days at 28°C and then cultured in potato dextrose broth (PDB) for 5-20 days under aerobic conditions at 150 rpm. The yeast strains used in our study are *Saccharomyces cerevisiae* BY4742 (YPH) (*MAT*α *his*3Δ1 *leu*2Δ0 *lys*2Δ0 *ura*3Δ0) (Stratagene, USA), INV*S*c1 (*MAT*α *his*3Δ1 *leu*2-3, 112 *trp*1-289 *ura*3-52) (Invitrogen), *S. cerevisiae* YSC2 (Type II, sigma) and *Candida albicans* SC5314. The yeast strains were grown at 30°C for 2-3 days cultured in rich YPD (2% glucose, 2% Bacto-peptone, 1% yeast extract) medium or minimal synthetic drop-out (SD) medium (2% glucose, 0.67% yeast nitrogen base, 0.5% ammonium sulphate with all appropriate amino acids, except uracil, leucine, or both depending upon the plasmid for selection). For the induction of the galactose regulated promoters, glucose was replaced with galactose as carbon source. For the cloning and propagation of yeast plasmids, the DH5α *E. coli* cells were cultured on the LB medium at 37°C.

### Phylogenetic analysis for gene selection

The putative cytochrome P450 gene sequences of *F. oxysporum* were obtained from the Fungal Cytochrome P450 Database [22]. Phylogenetic analysis was carried out with the putative *FoCYPs* and reported CYP52 P450s by neighbor-joining method using the Molecular Evolutionary Genetics Analysis tool (MEGA6) with the bootstrap value set to 1000. Multiple alignment was performed by using ClustalX program with the alignment parameters set to default. Sequence identity information was calculated by T-coffee software and BLAST (bl2seq) with the program set for highly similar sequences. Based on the *Fusarium* comparative database [16], the CPR gene of *F. oxysporum* (FOXG_08274) and its paralogues was selected and employed in our study.

### Extraction of genomic DNA, RNA and synthesis of cDNA

Fungal mycelia were harvested from 5, 10, 15 and 20-day-old cultures by vacuum filtration and frozen in liquid nitrogen. The frozen mycelia were completely ground to powder form using a mortar and pestle. The RNA was then extracted using the Qiagen RNeasy plant mini kit (Korea Ltd, Seoul) and stored at -80°C. The concentration of RNA was quantified at 260 nm using a Nanodrop (ND-1000 spectrophotometer; Thermo Fisher Scientific, DE, USA). An RNA cocktail was generated by mixing equal amounts of RNA isolated from the different days in the culture intervals. Using the RNA cocktail mixture, the first strand cDNA was synthesized with the QuantiTect reverse transcription kit, Qiagen (Hilden, Germany). The newly synthesized cDNA was stored at -20°C until PCR amplification of the *FoCYP* and *FoCPR* genes. To amplify the *ScCPR* and *CaCPR genes*, genomic DNA was extracted from the *S. cerevisiae*YSC2 and *C. albicans* SC5314 cells as described earlier [47].

### Construction of *FoCYP539A7* and *FoCYP655C2* reconstituted system in *S. cerevisiae*

PCR amplifications were carried out using custom designed oligonucleotides (Additional file 1: Table S3 and S4) obtained from Cosmo Genetech (Seoul, Korea). The templates for the *FoCYP* and *FoCPR* genes were *F. oxysporum* cDNA, and those for *ScCPR* and *CaCPR* genes were their respective genomic DNAs. PCR was carried out using LA Taq polymerase (Takara, Japan). The annealing temperature of 54°C was used for the *FoCYP539A7* (FOXG_00101) and *FoCYP655C2* (FOXG_14594) genes, 61°C was used for *FoCPR* and *ScCPR* genes, and 59°C for the *CaCPR* gene. The *FoCYP* genes were cloned into the pESC_URA vector (Stratagene, USA), and the *FoCPR, ScCPR* and *CaCPR* genes were ligated into the pESC_LEU vector (Stratagene, USA) using the *SpeI* and *SacI* restriction enzymes with the T4 DNA ligase enzyme (NEB, MA, USA). The ligated products were transformed into DH5α *E.coli* cells and selected on LB agar medium containing 100 μg/mL ampicillin. The positive transformants were selected by colony PCR and restriction digestion of the cloned plasmids. The recombinant plasmids harboring the cloned genes were further confirmed by gene sequencing (Cosmo Genetech, Korea). Yeast transformations were carried out into the *S. cerevisiae* BY4742cells using the lithium acetate method described previously [48]. The pESC_URA plasmids harboring the *FoCYP* genes were transformed individually (control) and also co-transformed with the pESC_LEU plasmids harboring *ScCPR, CaCPR* and *FoCPR*. The positive transformants were selected on the minimal SD agar medium. For further confirmation of the positive transformants, plasmids were extracted from the transformed yeast cells and PCR reactions were carried out using the gene specific primers.

### Isolation of microsomes and CO difference spectral analysis

A single yeast colony harboring *FoCYP539A7* and *FoCYP655C2* gene was individually inoculated into 10 mL of SD-U (except uracil) medium with 2% dextrose. *S. cerevisiae* harboring only pESC_URA plasmid without any *FoCYP* was used as control. The overnight grown cells were inoculated into 50 mL of YPG media with 4% galactose and 2 mM 5-ALA to obtain an OD_600_ of 0.4 and cultured again. The cells were collected, resuspended in 500 mL of fresh galactose media, and cultured for about 2 days with shaking at 150 rpm until reaching an OD_600_ of 2-4. The galactose induced yeast cells were then harvested and the microsomes were isolated as described earlier [49]. UV absorbance spectra of CO-bound microsomes after sodium dithionate reduction were recorded using a UV-visible spectrophotometer (Thermo Labsystems, NY, USA) scanning between the wavelengths 400 and 500 nm.

### Inactivation of POX1 gene in *S. cerevisiae*

PCR-mediated gene disruption was carried out to inactivate the acyl-CoA oxidase (pox1 gene) from *S. cerevisiae* INV*S*c1 cells. Oligonucleotides (Additional file 1: Table S3) were designed to amplify the *Schizosaccharomyces pombe his5*^*+*^ gene (which complements *S. cerevisiae his3* mutations) with 40 bp the flanking region on either side that had homology to the flanking region of *pox1*. PCR reactions were carried out using Han-pfu polymerase (Genenmed Inc., Korea) with the template DNA as plasmid pFA6a-His3MX6 [50], and the annealing temperature was set to 55-68°C. The pFA6a plasmid containing *P*_*TEF*_-*his5*^+^-*T*_*TEF*_ fragment was cloned using the *BamHI* and *EcoRI* restriction enzymes with the T4 DNA ligase enzyme (NEB, MA, USA). The PCR product was purified and ~1.0 μg of DNA was used for transformation into *S. cerevisiae* as described previously [50]. The selection for histidine prototrophs (transformants) was carried out on the SD medium containing adenine and the appropriate amino acids except histidine.

### Functional analysis of *FoCYP539A7 and FoCYP655C2* reconstituted systems

Double transformations were carried out into the ΔPOX1 mutant *S. cerevisiae* to express both the *FoCYP* and CPR genes together. Hence, the pESC_URA plasmids harboring the *FoCYP* genes were co-transformed with the pESC_LEU plasmids harboring *ScCPR / CaCPR / FoCPR* and selected on the SD-U,-L,-H medium (except uracil, leucine and histidine). For control experiments, *S. cerevisiae* cells harboring only *FoCYP*s without any CPR were employed. Functional analysis of *FoCYP539A7* and *FoCYP655C2* was initially carried out in an *in vitro* system and a resting cell system. Later on, a biotransformation system was employed for the sustainable production of omega hydroxy fatty acids in yeast system. A single colony of yeast reconstituted system harboring both the *FoCYP* and CPR genes were cultured in 10 mL of SD-U,-L,-H medium with 2% dextrose and cultured at 30°C. The overnight grown cells were then inoculated into 500 mL of SD or YPG media with 4% galactose and 2 mM 5-ALA and cultured as described above. For the *in vitro* system, microsomes were isolated from all the reconstituted systems of *S. cerevisiae* cells as described earlier. The *in vitro* reaction was performed with the standard assay mixture containing 50 μg/mL of microsomal proteins, 100 μM potassium phosphate buffer (pH 7.0), 500 μM NADPH and 100 μM of substrates (lauric acid, myristic acid and palmitic acid) and incubated at 30°C for 30 minutes with shaking at 150 rpm. The products were then extracted with equal volumes of ethyl acetate, dried in a vacuum concentrator and converted to their trimethylsilyl (TMS) derivatives by incubating at 50°C for 20 minutes with BSTFA and analyzed by gas chromatography (GC). In the resting cell system, the galactose induced cells were harvested by centrifugation (3500 rpm, 10 min, 4°C), washed once with 25 ml of 100 mM Tris-HCl or potassium phosphate buffer, and then resuspended in 25 ml of 100 mM Tris-HCl or potassium phosphate (pH 7.5) buffer. 100 μM of C12, C14 and C16 substrates were added to the reaction mixture and the cells were incubated at 30°C for 24 hours with shaking at 150 rpm. In the biotransformation system, the overnight grown cells were then inoculated into 25 mL of SD or YPG media with 4% Galactose and 2 mM 5-ALA to obtain an OD_600_ of 0.4 and further cultured until the cells reached an OD_600_ of 1.0-1.2. The cells were then harvested and resuspended in fresh galactose media and 500 μM of substrates: C6-C16 fatty acids were added and the cells were cultured again for 48-72 hrs. The pH of the growing yeast cell cultures were maintained at pH 7.0 and pH 5.5 for the biotransformation reactions. The reaction products were collected at different time intervals, acidified with 6 M HCL to *ca*. pH 2 and extracted with equal volumes of ethyl acetate by vigorous vortexing and centrifugation at 14000 rpm. The reaction metabolites were then dried in the concentrator, dissolved in ethyl acetate and derivatized with BSTFA as described above. The derivatized metabolites were then analyzed by gas chromatography (GC) and mass spectrometry (MS).

### Product identification and quantification

Quantitative analysis of derivatized metabolites was performed in a GC HP 6890Series (Agilent Technologies, USA) equipped with a flame ionization detector (GC/FID). The sample (2 μL) was injected by split mode (split ratio 20.0:1) and analyzed using a non-polar capillary column (5% phenyl methyl siloxane capillary 30 m × 320 μm i.d., 0.25 μm film thickness, HP-5). The oven temperature program was: 50°C for 1 minute, increase by 15°C/min to 250°C and hold for 10 minutes. The inlet temperature was 250°C and for the detector, it was 280°C. The flow rate of the carrier gas (He) was 1 mL/min, and the flow rates of H_2_, air, and He in FID were 45 mL/min, 400 mL/min, and 20 mL/min respectively. The peaks were identified by comparison of GC chromatograms with those of authentic references.

Qualitative analysis of derivatized metabolites was performed by GC/MS using a TRACE GC ULTRA gas chromatograph (Thermo Scientific, USA), which was coupled to an ion trap mass detector ITQ1100 (Thermo Scientific, USA). The reaction sample (1 μL) was injected by splitless mode (0.8 minute of splitless time) and analyzed using a non-polar capillary column (5% phenyl methyl siloxane capillary 30 m × 250 μm i.d., 0.25 μm film thickness, TR-5 ms). The oven temperature program was: 50°C for 1 minute, increase by 15°C/min to 250°C and hold for 10 minutes. The temperatures for inlet, mass transfer line and ion source were 250°C, 275°C, and 230°C, respectively. The flow rate of the carrier gas (He) was 1.0 mL/min, and the electron energy for the EI mass spectrum was 70 eV. The mass spectral peaks were identified by comparison of retention times and mass spectral data of the reaction sample with those of authentic references.

### Determination of expression level of CPRs by MTT assay

The expression levels of *ScCPR* and *FoCPR* were analyzed by 3-(4,5-dimethylthiazol-2-yl)-2,5-diphenyl tetrazolium bromide (MTT) assay based on its reductase activity. Microsomes were isolated from the *S. cerevisiae* cells harboring CYP539A7-ScCPR, CYP539A7-FoCPR, CYP655C2-ScCPR and CYP655C2-FoCPR reconstituted systems. The concentrations of the total isolated microsomal proteins were calculated based on the bradford assay and the expression level of *FoCYP539A7* and *FoCYP655C2* in all the reconstituted systems were subsequently estimated by CO binding analysis. For the MTT reductase assay, the microsomal concentrations for all the reconstituted systems were normalized to 10 μg/mL. MTT reductase activity was carried out with 100 μM MTT, 10 μg/mL microsomes in 100 mM potassium phosphate buffer (pH 7.6), and the reaction was initiated following the addition of 100 μM NADPH [39]. The change in absorbance was measured at 610 nm using a UV-visible spectrophotometer (Thermo Labsystems, NY, USA) and an extinction coefficient of 11.3 mM^−1^ cm^−1^ was used to calculate the number of moles of MTT reduced.

### Molecular modeling studies

From the Fungal Cytochrome P450 Database, the translated gene sequences of *FoCYP539A7* and *FoCYP655C2* were retrieved and modeled using Modeler [51]. Prior to the modeling study, a protein blast search was performed against Protein structural databank (PDB) for the protein sequences *FoCYP539A7* and *FoCYP655C2*. While modeling the protein structures, the heme from the templates were also imported using Modeler-Ligand import option. Further, the stereo chemical quality of the model was validated using SAVES server. Later, the ligand binding sites were predicted for the key residues responsible for the hydrogen bond interaction with the carbonyl oxygen of the fatty acid substrates. To identify the key residues, a flexible docking study of modeled structures with its best substrate capric acid was carried out. Correspondingly, molecular docking calculations were done for the 3D structures of the fatty acids - capric, caprylic, lauric and myristic acid with their respective modeled structure using GOLD [52]. As the fatty acid contains increased number of rotatable bonds, it can take huge number of conformation while docking in the active site. For that reason, we retrieved the different conformers of the fatty acids [caprylic (Scid-379), capric (Scid-2969), lauric (Scid-3893) and myristic acid (Scid-11005)] from the Pubchem substance database. The ionization states of the fatty acids were generated using Accelrys Discovery Studio 2.1 (DS2.1; accelrysInc., CA, USA). Finally, the best docked complexes of the fatty acids showing hydrogen bond interactions with *FoCYP539A7* and *FoCYP655C2* were exported and compared for further analysis using pymol [53].

## Additional file

Additional file 1: Table S1. Final yield (mg/L) of ω-hydroxy fatty acids by FoCYPs with the heterologous (ScCPR) and homologous (FoCPR) reductases. **Table S2.** Active site amino acids residing 5Å of capric acid docked complexes of FoCYPs. **Table S3.** Oligonucleotide primers used for gene amplification and yeast expression. **Table S4.** Oligonucleotide primers employed in this study. **Figure S1.** Phylogenetic analysis of 169 putative FoCYPs with the reported ω-selective or specific fatty acid CYPs. **Figure S2.** Multiple sequence alignment of FoCYP539A7 and FoCYP655C2 with the reported ω-selective or specific fatty acid CYPs. **Figure S3.** Construction of yeast expression vector. **Figure S4.** Alternative oxidation pathways for fatty acids in yeast. **Figure S5.** PCR confirmation of POX1 disruption. **Figure S6.** Determination of expression levels of P450 and CPR in the heterologous and homologous reconstituted systems. **Figure S7.** Reaction profile of hydroxylation of fatty acids by FoCYP539A7 and FoCYP655C2 in the biotransformation carried out at pH 5.5. **Figure S8** Final yield (mg/L) of ω-hydroxy fatty acids by FoCYP539A7 and FoCYP655C2 with the heterologous reductase (ScCPR) in the biotransformation carried out at pH 5.5 and pH 7.0. **Figure S9.** Representative Gas chromatographic analysis patterns of omega hydroxylated products by FoCYP539A7 and FoCYP655C2 reconstituted system. **Figure S10.** Representative Mass Spectral analysis patterns of omega hydroxylated products by FoCYP539A7 and FoCYP655C2 reconstituted system. **Figure S11.** (A) Homology modeled structure of FoCYP539A7 using 2Q9F as template. (B) Superimposed structure of model structure (cyan) and template structure -2Q9F (red). **Figure S12.** Ramachandran plot for modeled FoCYP539A7 derived from homology modeling. Figure S13 (A) Homology modeled structure of FoCYP655C2 using 1TQN as template. (B) Superimposed structure of model structure (cyan) and template structure -1TQN (red). **Figure S14.** Ramachandran plot for modeled FoCYP655C2 derived from homology modeling.

## Abbreviations

FoCYP: *Fusarium oxysporum* cytochrome P450
CPR: Cytochrome P450 reductase
FA: Fatty acid
ω-OHFA: Omega hydroxy fatty acid
ΔPox1: Pox1 deletion
