# Supplementary material for: Fungal cytochrome P450 monooxygenases of Fusarium oxysporum for the synthesis of ω-hydroxy fatty acids in engineered Saccharomyces cerevisiae
Source: Microb Cell Fact. 2015 Apr 2;14:45. doi: 10.1186/s12934-015-0228-2 (PMC4387584; doi:10.1186/s12934-015-0228-2)
Supplement: Additional file 1: Table S1. — Final yield (mg/L) of ω-hydroxy fatty acids by FoCYPs with the heterologous (ScCPR) and homologous (FoCPR) reductases. Table S2. Active site amino acids residing 5Å of capric acid docked complexes of FoCYPs. Table S3. Oligonucleotide primers used for gene amplification and yeast expression. Table S4. Oligonucleotide primers employed in this study. Figure S1. Phylogenetic analysis of 169 putative FoCYPs with the reported ω-selective or specific fatty acid CYPs. Figure S2. Multiple sequence alignment of FoCYP539A7 and FoCYP655C2 with the reported ω-selective or specific fatty acid CYPs. Figure S3. Construction of yeast expression vector. Figure S4. Alternative oxidation pathways for fatty acids in yeast. Figure S5. PCR confirmation of POX1 disruption. Figure S6. Determination of expression levels of P450 and CPR in the heterologous and homologous reconstituted systems. Figure S7. Reaction profile of hydroxylation of fatty acids by FoCYP539A7 and FoCYP655C2 in the biotransformation carried out at pH 5.5. Figure S8 Final yield (mg/L) of ω-hydroxy fatty acids by FoCYP539A7 and FoCYP655C2 with the heterologous reductase (ScCPR) in the biotransformation carried out at pH 5.5 and pH 7.0. Figure S9. Representative Gas chromatographic analysis patterns of omega hydroxylated products by FoCYP539A7 and FoCYP655C2 reconstituted system. Figure S10. Representative Mass Spectral analysis patterns of omega hydroxylated products by FoCYP539A7 and FoCYP655C2 reconstituted system. Figure S11. (A) Homology modeled structure of FoCYP539A7 using 2Q9F as template. (B) Superimposed structure of model structure (cyan) and template structure -2Q9F (red). Figure S12. Ramachandran plot for modeled FoCYP539A7 derived from homology modeling. Figure S13 (A) Homology modeled structure of FoCYP655C2 using 1TQN as template. (B) Superimposed structure of model structure (cyan) and template structure -1TQN (red). Figure S14. Ramachandran plot for modeled FoCYP655C2 derived from homology modeling. [file 12934_2015_228_MOESM1_ESM.docx]

**Fungal cytochrome P450 monooxygenases of *Fusarium oxysporum* for the synthesis of ω-hydroxy fatty acids in engineered *Saccharomyces cerevisiae***

Pradeepraj Durairaj^a^, Sailesh Malla^b#^, Saravanan Prabhu Nadarajan^c^, Pyung-Gang Lee^b^, Eunok Jung^b^, Hyun Ho Park^a^, Byung-Gee Kim^b^ and Hyungdon Yun^c*^

^a^ School of Biotechnology, Yeungnam University, Gyeongsan, South Korea.

^b^ School of Chemical and Biological Engineering, Seoul National University, Seoul, South Korea.

^c^ Department of Bioscience and Biotechnology, Konkuk University, Seoul, South Korea.

^#^ Current position: Novo Nordisk Foundation Center for Biosustainability, Technical University of Denmark, Denmark.

E-mail: [hyungdon@konkuk.ac.kr](mailto:hyungdon@konkuk.ac.kr); [Tel]: (+82)-2-4500496

^*^ Corresponding author

**Table S1 Final yield (mg/L) of ω-hydroxy fatty acids by *FoCYP*s with the heterologous (*ScCPR*) and homologous (*FoCPR*) reductases.**

| **Reconstituted system** | | **8-hydroxyoctanoic acid** | | **10-hydroxydecanoic acid** | | **12-hydroxydodecanoic acid** | |
| --- | --- | --- | --- | --- | --- | --- | --- |
|  |  | **pH-7.0** | **pH-5.5** | **pH-7.0** | **pH-5.5** | **pH-7.0** | **pH-5.5** |
| CYP539A7 | FoCPR | 38.2 | 45.1 | 63.1 | 73.8 | 60.4 | 72.2 |
|  | ScCPR | 27.6 | 36.2 | 48.6 | 57.8 | 46.0 | 56.8 |
| CYP655C2 | FoCPR | - | - | 41.4 | 52.2 | 39.4 | 51.9 |
|  | ScCPR | - | - | 29.0 | 38.5 | 27.0 | 36.0 |

**Table S2 Active site amino acids residing 5Å of capric acid docked complexes of *FoCYP*s.**

| **CYP** | **Active site residues** | **Key residue**  **(Hydrogen bond interaction)** |
| --- | --- | --- |
| ***FoCYP539A7*** | Leu107, Phe391, Leu111, Ile110, Ile516, Val510, Pro390, Leu322, Thr328, Alal324, Val389, Phe137, Asn392 | Asn106 |
| ***FoCYP655C2*** | Leu240, Phe121, Phe118, Phe136, Leu386, Thr134, Ala134, Asn382, Thr323, Ala319 | Arg235 |

**Table S3** Oligonucleotide primers used for gene amplification and yeast expression.

| **S.No** | **OLIGO NAME** | **PRIMER SEQUENCE 5’to 3’** | **DETAILS** |
| --- | --- | --- | --- |
| 1 | FoCYP539A7 FWD | ACAC*ACTAGT*ATGGGTGTCGTGGAAGCTCTT | Cloned into pESC-URA vector using SpeI and SacI restriction enzymes. |
|  | FoCYP539A7 REV | GAGA*GAGCTC*TTAATTCTGGGCTTCCCAGAA |  |
| 2 | FoCYP655C2 FWD | GAGA*ACTAGT*ATGGCGCTCCATCAGTTACTC |  |
|  | FoCYP655C2 REV | AGAG*GAGCTC*TTATCTGTATAGAGCCACCTT |  |
| 3 | ScCPR FWD | AAAA*ACTAGT*ATGCCGTTTGGCATTGATAAC | Cloned into pESC-LEU vector using SpeI and SacI restriction enzymes. |
|  | ScCPR REV | AAAA*GAGCTC*TTATTACCACACATCTTCCTGAT |  |
| 4 | CaCPR FWD | AAAA*ACTAGT*ATGGCATTAGACAAATTAGATTT |  |
|  | CaCPR REV | AAAA*GAGCTC*TTACCAAACATCTTCTTGATATC |  |
| 5 | FoCPR FWD | ATAT*ACTAGT*ATGGCTGAACTCGACACTCTG |  |
|  | FoCPR REV | ATAT*GAGCTC*CTATGACCAAACATCCTCCTG |  |
| 6 | Pox1_DelFWD | TCACAGAAAAAAAGAAAATATAATAAATTAGTATTGCGATGTCACCCGGCCAGCGACATG | pFA6a plasmid containing *P_TEF_*-*his5*^+^-*T_TEF_* fragment cloned into the *BamHI* and *EcoRI* sites. |
|  | Pox1_Del REV | CGCAAAACAGAGGGTTCGAAGGAAAACAGGAAACCTCTACGATATTACTTTCTGCGCACT |  |

Restriction sites introduced into the gene specific primers for cloning are indicated in *italics.* ScCPR –*Saccharomyces cerevisiae* CPR, CaCPR – *Candida albicans* CPR, FoCPR – *Fusarium oxysporum* CPR. The underlined regions correspond to *his5+* sequences, and the remaining sequences are the flanking residues of *pox1.*

**Table S4** Oligonucleotide primers employed in this study.

| **S.No** | **OLIGO NAME** | **PRIMER SEQUENCE 5’to 3’** | **DETAILS** |
| --- | --- | --- | --- |
| 1 | FOXG_03506 FWD | GCGC*ACTAGT*ATGATGGATGTCTCTACGGAA | Partial length P450 |
|  | FOXG_03506 REV | GAGA*GAGCTC*TTACCTCTTGGCTGCCACTCG |  |
| 2 | FOXG_03951 FWD | GCGC*ACTAGT*ATGACAGGCTTTATCGCCGATTTA | Not amplified |
|  | FOXG_03951 REV | AGAG*GAGCTC*TTAATGCAGCAACTACTCTCT |  |
| 3 | FOXG_14589 FWD | GAGA*ACTAGT*ATGTCATCTCGTCTCTCCGCG |  |
|  | FOXG_14589 REV | ATAT*GAGCTC*TTACCTCGGGGTGACAGCGAT |  |
| 4 | FOXG_10811 FWD | GAGA*ACTAGT*ATGTCTATCTTAGGCACCACC |  |
|  | FOXG_10811 REV | GAAG*GAGCTC*TTAAGCCAGCTTCAACTGGCA |  |

Restriction sites introduced into the gene specific primers for cloning are indicated in *italics.*

**Figure S1 Phylogenetic analysis of 169 putative *FoCYP*s with the reported ω-selective or specific fatty acid CYPs.** FOXG_10811, FOXG_14589, FOXG_14594, FOXG_03951, FOXG_03506 and FOXG_00101 *FoCYP*s are clustered together along with the same gene cluster of CYP52 candidates. CYP52A3, CYP52A4, CYP52A5 and CYP52A9 are from *Candida maltosa*, CYP52A17 and CYP52A21 are from *C. albicans*, and CYP52A13 is from *C. tropicalis*. Phylogenetic analysis was performed by Neighbor-joining method using the Molecular Evolutionary Genetics Analysis tool (MEGA6) with the bootstrap value set to 1000.

**
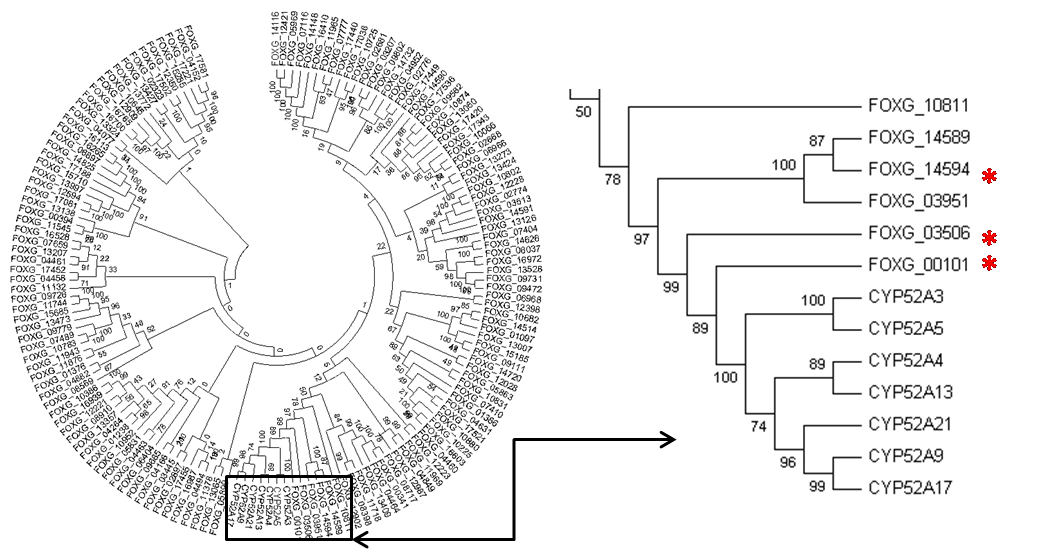
**

**Figure S2** **Multiple sequence alignment of *FoCYP539A7* and *FoCYP655C2* with the reported ω-selective or specific fatty acid CYPs.** The amino acid residues corresponding to the heme binding domain is marked with a dotted box. Multiple alignment was performed using ClustalX program with the alignment parameters set to default. *FoCYP539A7* and *FoCYP655C2* are from *F. oxysporum,* CYP52A3, CYP52A4, CYP52A5 and CYP52A9 are from *Candida maltosa*, CYP52A17 and CYP52A21 are from *C. albicans*, and CYP52A13 is from *C. tropicalis*.


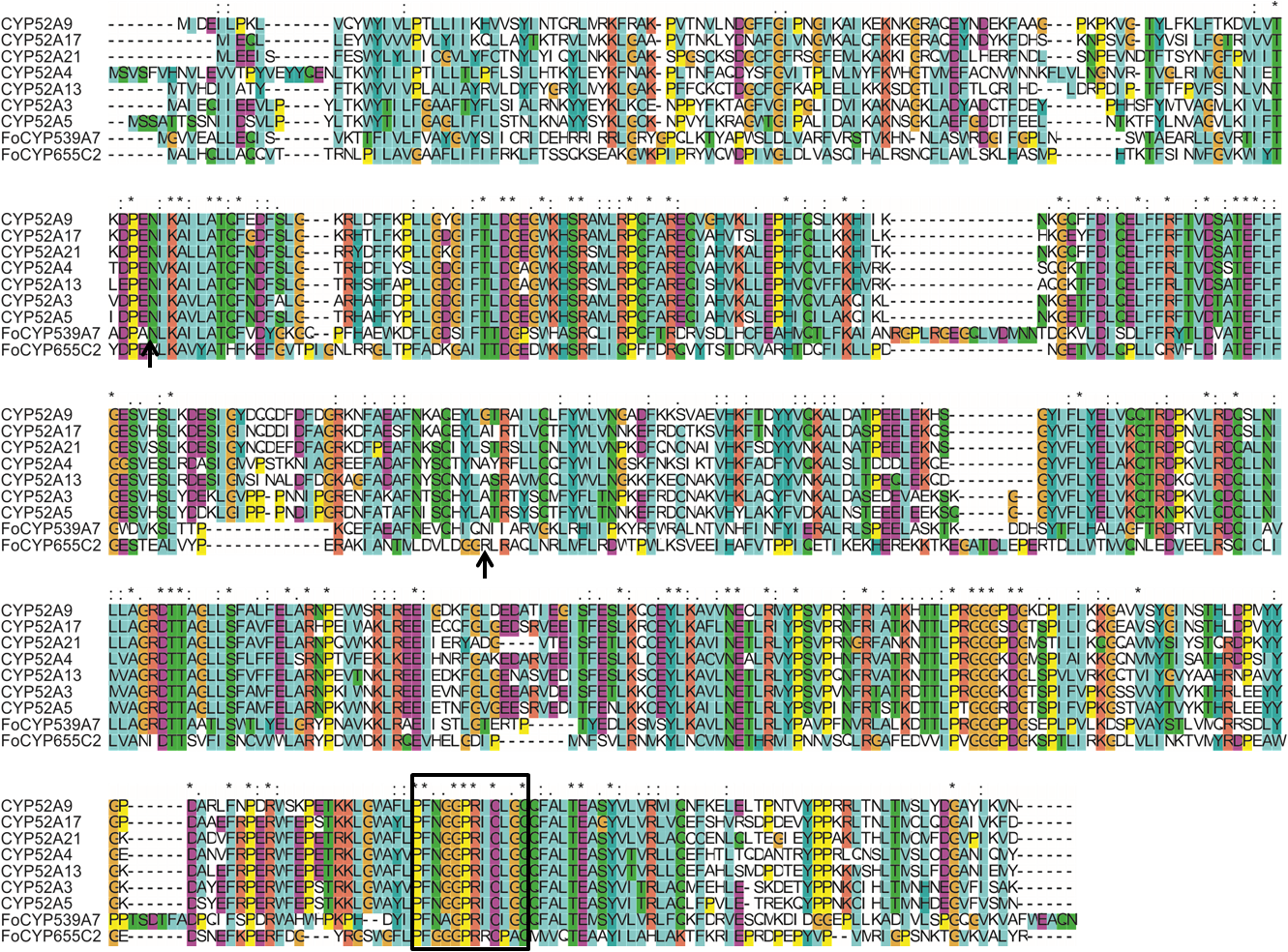


**Figure S3** **Construction of yeast expression vector** **(A)** pESC-URA vector harboring P450 (*FoCYP*), **(B)** pESC-LEU vector harboring CPR (*ScCPR / CaCPR /FoCPR*).

**
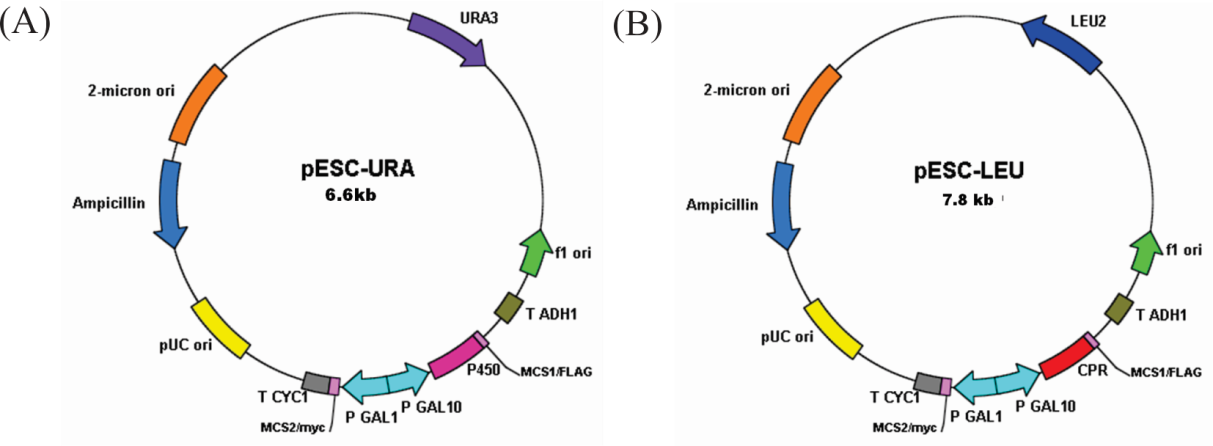
**

**Figure S4 Alternative oxidation pathways for fatty acids in yeast.** ω-oxidation is an alternative pathway to β-oxidation, which becomes more important when β oxidation is defective [2, 32].

**Figure S5 PCR confirmation of POX1 disruption.** T – HisMX transformant of *S. cerevisiae* INVSc1; DC - Double crossover mutant (*S. cerevisiae* ΔPOX); C - *S.cerevisiae* INvSc1 (control strain); M - DNA marker.

**Figure S6 Determination of expression levels of P450 and CPR in the heterologous and homologous reconstituted systems. (A)** CO binding analysis of microsomes of CYP539A7-FoCPR and CYP539A7-ScCPR. **(B)** CO binding analysis of microsomes of CYP655C2-FoCPR and CYP655C2-ScCPR.Yeast expression was carried out using 4% galactose, 2 mM 5-ALA at 30 ºC. **(C, D)** MTT reductase assay to verify the expression level of CPRs. 10 µg/mL microsomes of each CYP-CPR reconstituted systems were treated with MTT and the color change was observed following the addition of NADPH. The reduction of MTT into blue formazon was measured at 610 nm and an extinction coefficient of 11.3 mM^−1^cm^−1^ was used to calculate the number of moles of MTT reduced.

**
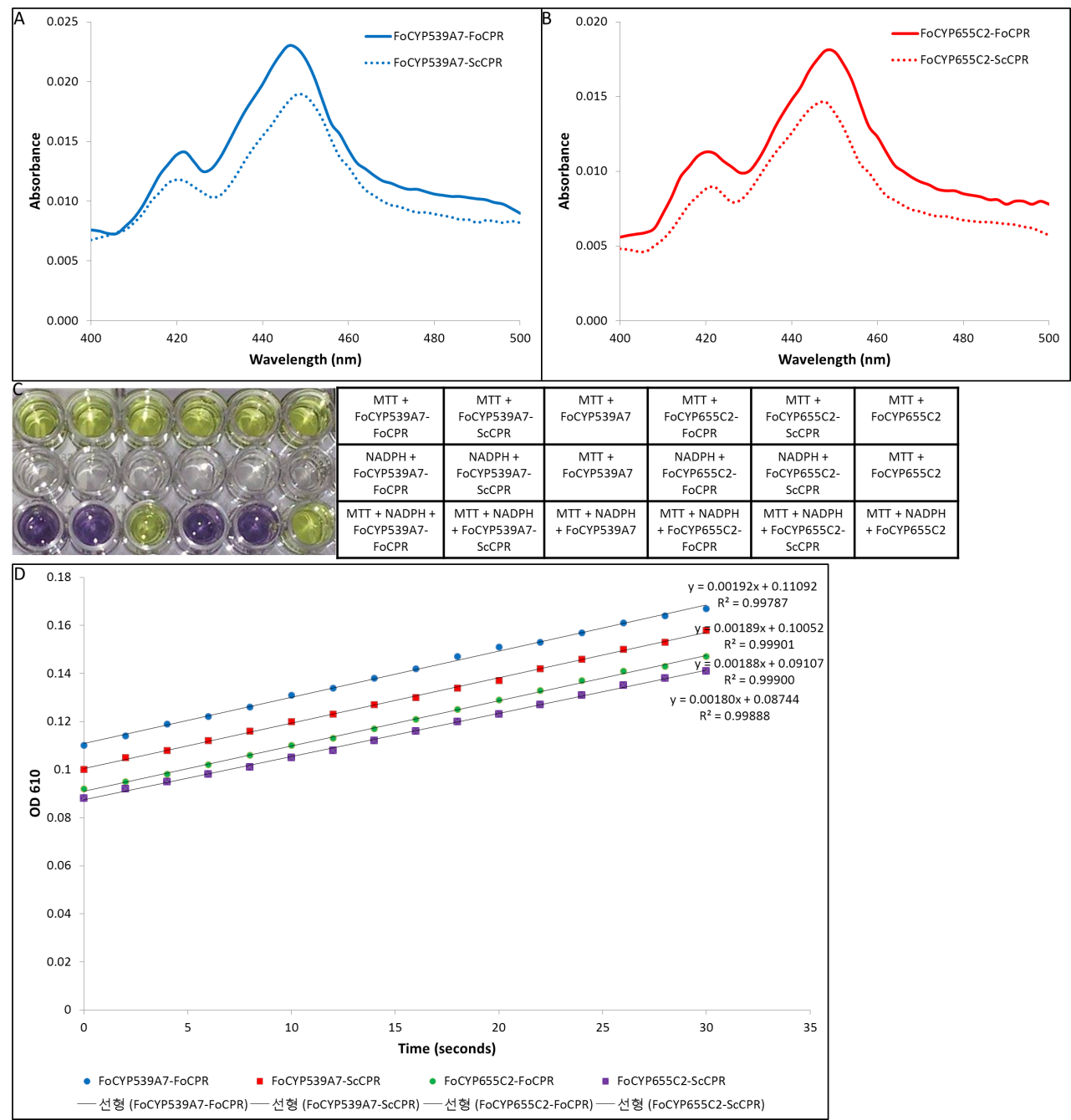
**

**Figure S7 Reaction profile of hydroxylation of fatty acids by *FoCYP539A7* and *FoCYP655C2* in the biotransformation carried out at pH 5.5. (A)** Homologous reconstituted systems (with *FoCPR*). **(B)** Heterologous reconstituted systems (with *ScCPR*). ΔPox1 mutant S. cerevisiae cells harboring the CYP39A7-ScCPR, CYP655C2-ScCPR, CYP539A7-FoCPR and CYP655C2-FoCPR reconstituted systems were induced with 4% galactose, 2 mM 5-ALA and 500 µM of substrates: caprylic acid (C8), capric acid (C10) and lauric acid (C12) were added and cultured at pH 5.5. Samples collected at 10 hr intervals were extracted, trimethylsilyl derivatized and analyzed by GC.


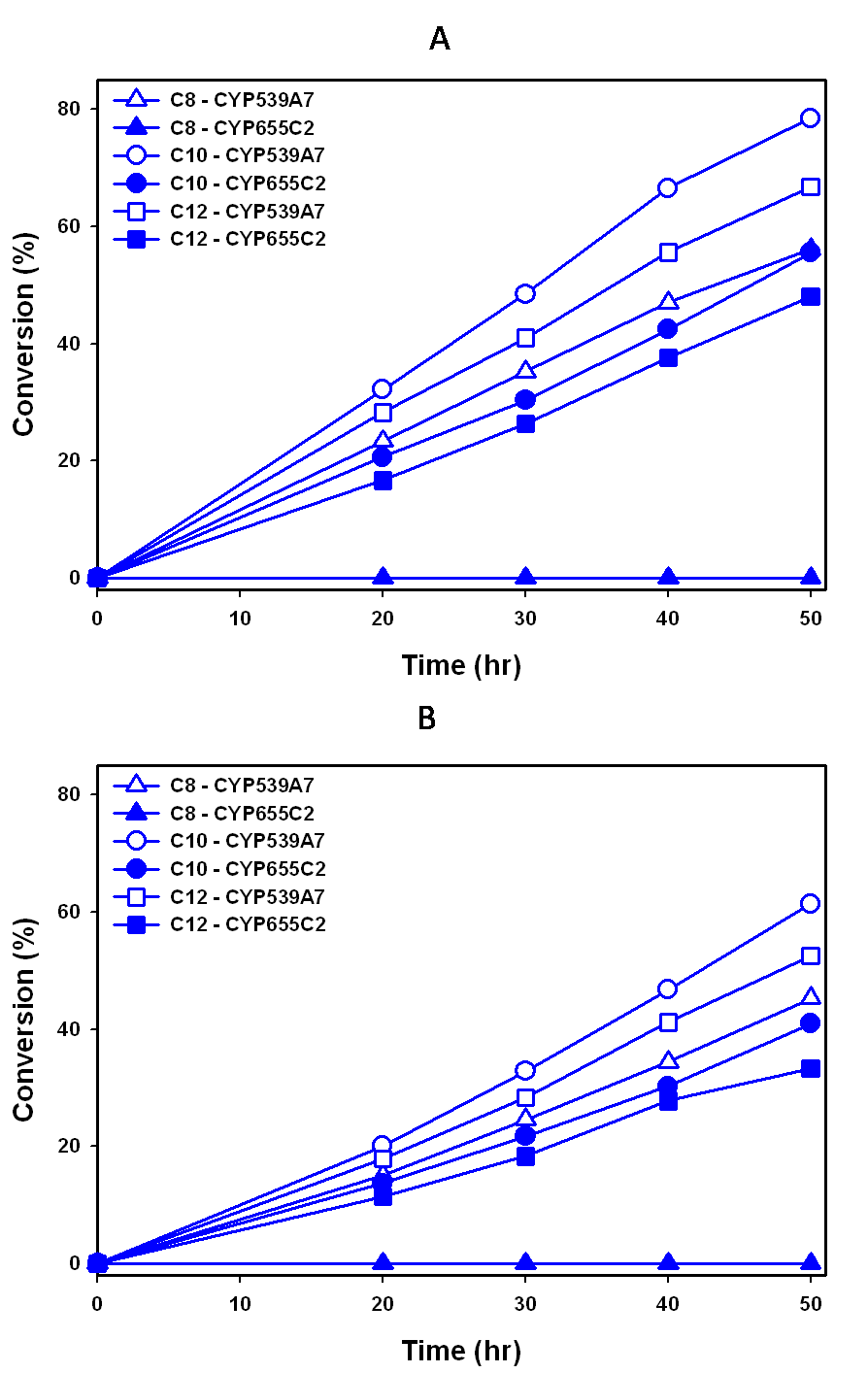


**Figure S8 Final yield (mg/L) of ω-hydroxy fatty acids by *FoCYP539A7* and *FoCYP655C2* with the heterologous reductase (*ScCPR*) in the biotransformation carried out at pH 5.5 and pH 7.0.** Data were plotted from the 50 hr biotransformation reaction samples. ΔPox1 mutant S. cerevisiae cells harboring the CYP539A7-ScCPR and CYP655C2-ScCPR reconstituted systems were induced with 4% galactose, 2 mM 5-ALA and 500 µM of substrates: caprylic acid (C8), capric acid (C10) and lauric acid (C12) were added and cultured at pH 5.5 and pH 7.0. Samples collected at 10 hr intervals were extracted, trimethylsilyl derivatized and analyzed by GC.


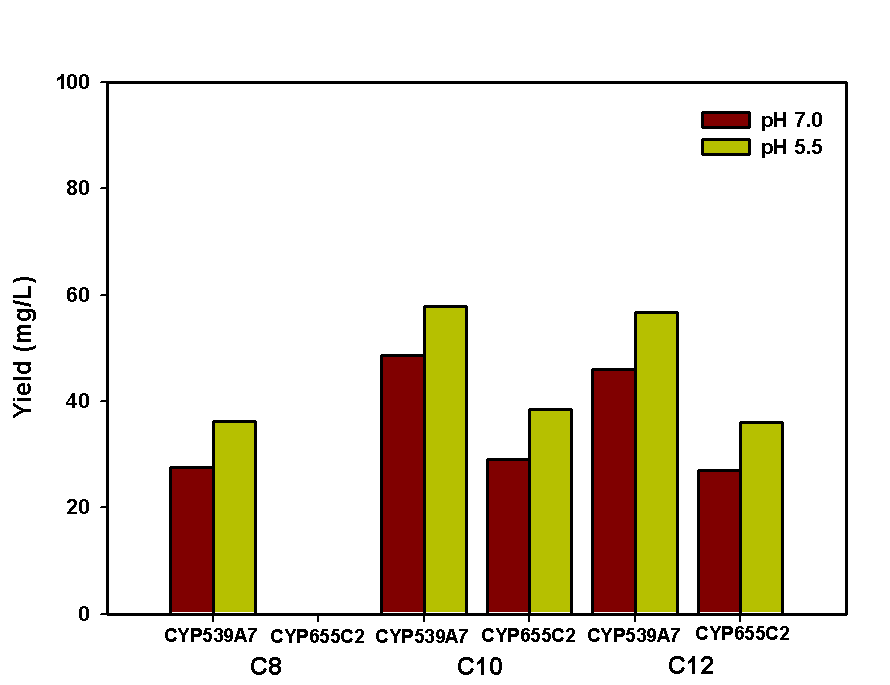


**Figure S9 Representative Gas chromatographic analysis patterns of omega hydroxylated products by *FoCYP539A7* and *FoCYP655C2* reconstituted system.** The retention times of reaction metabolites acid were identical to that of respective standard reference compounds. GC analysis was performed after derivatizing the reaction metabolites and standard compounds with BSTFA. **(A)** C8 standards and biotransformation samples, **(B)** C10 standards and biotransformation samples, **(C)** C12 standards and biotransformation samples

**
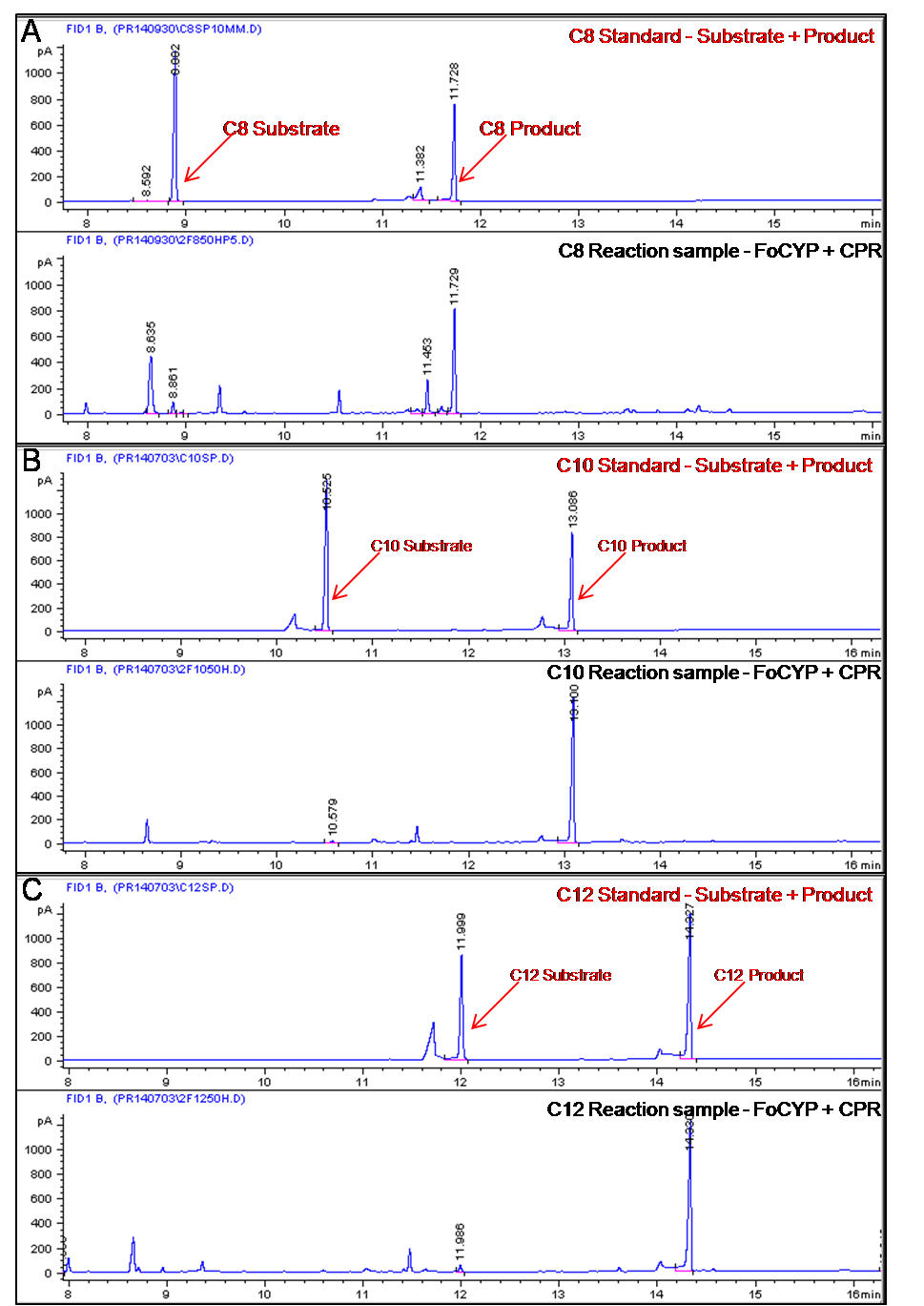
**

**Figure S10 Representative Mass Spectral analysis patterns of omega hydroxylated products by *FoCYP539A7* and *FoCYP655C2* reconstituted system. (A)** A major metabolite of hydroxylated caprylic acid displayed a mass spectrum with prominent ions at m/z 306, 290, 274 and was identified as 8-hydroxyoctanoic acid, **(B)** A major metabolite of hydroxylated capric acid showed a mass spectrum with prominent ions at m/z 333, 318, 302 and was identified as 10-hydroxydecanoic acid, **(C)** A major metabolite of hydroxylated lauric acid showed a mass spectrum with prominent ions at m/z 361, 346, 330 and was identified as 12-hydroxydodecanoic acid. The MS patterns of the reaction metabolites were found to be identical to the respective standard compounds. GCMS analysis was performed after derivatizing the reaction metabolites and standard compounds with BSTFA.

**

**

**Figure S11 (A)** Homology modeled structure of FoCYP539A7 using 2Q9F as template. Secondary structures are represented as Red helix, yellow beta strand and green loops. **(B)** Superimposed structure of model structure (cyan) and template structure -2Q9F (red).

**
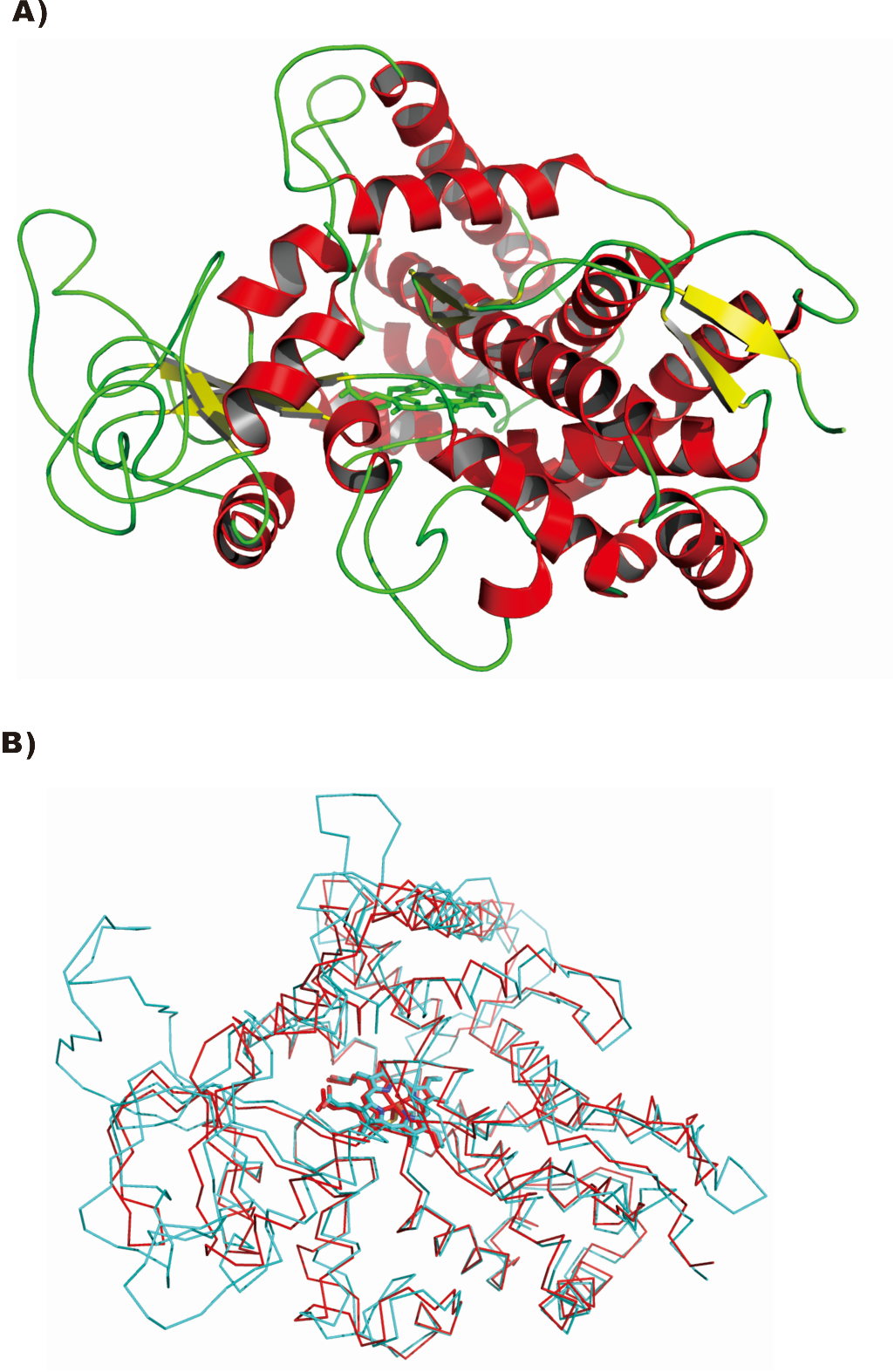
**

**Figure S12 Ramachandran plot for modeled *FoCYP539A7* derived from homology modeling.**

**Figure S13 (A)** Homology modeled structure of *FoCYP655C2* using 1TQN as template. Secondary structures are represented as Red helix, yellow beta strand and green loops. **(B)** Superimposed structure of model structure (cyan) and template structure -1TQN (red).

**
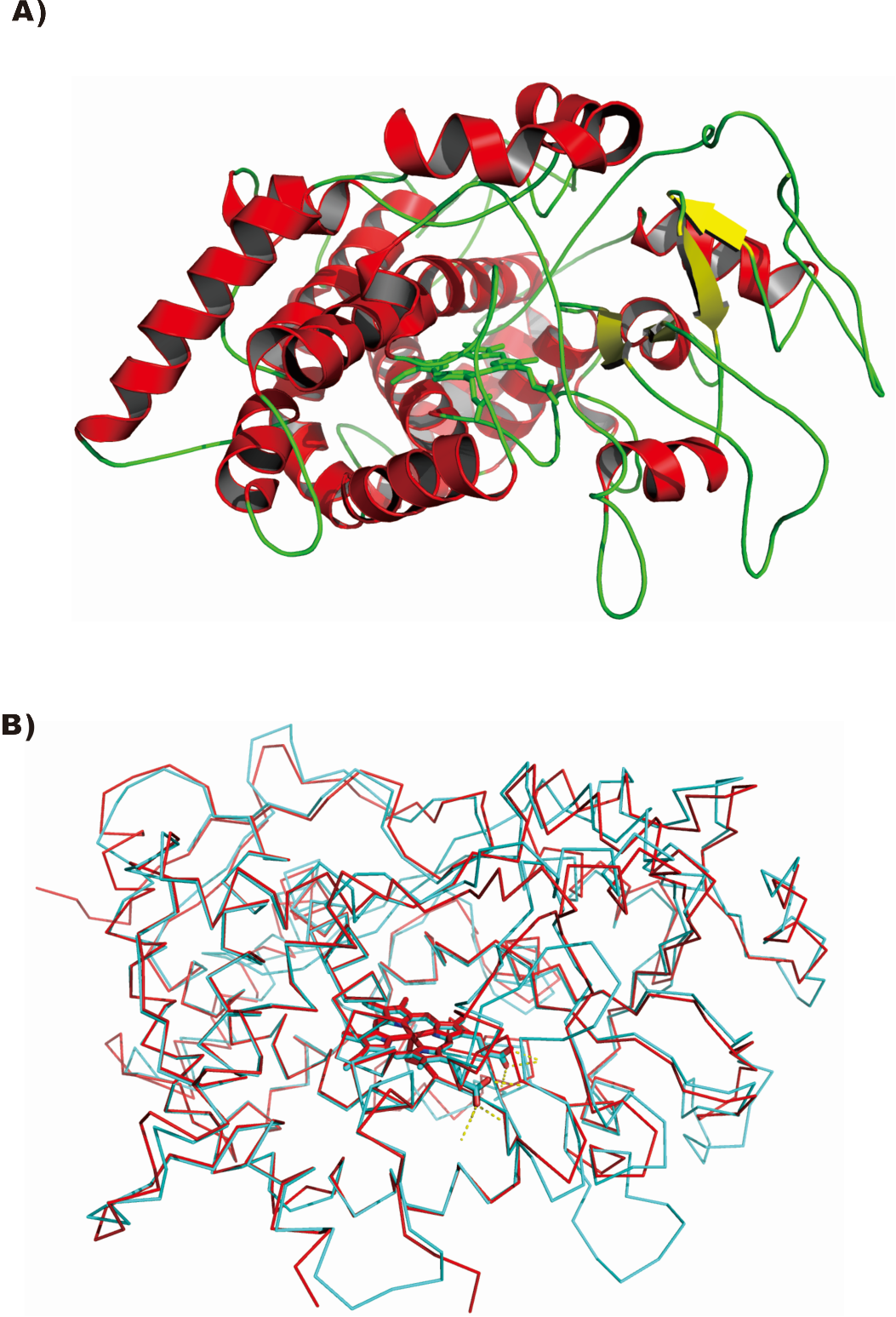
**

**Figure S14 Ramachandran plot for modeled *FoCYP655C2* derived from homology modeling**. Since the template (2Q9F) itself shows 84.7% favored region, we have got 81.5% residues in most favored region.
